# An integrated, cross-regulation pathway model involving activating/adaptive and feed-forward/feed-back loops for directed oscillatory cAMP signal-relay/response during the development of *Dictyostelium*

**DOI:** 10.3389/fcell.2023.1263316

**Published:** 2024-01-31

**Authors:** Pundrik Jaiswal, Netra Pal Meena, Fu-Sheng Chang, Xin-Hua Liao, Lou Kim, Alan R. Kimmel

**Affiliations:** ^1^ Laboratory of Cellular and Developmental Biology, National Institute of Diabetes and Digestive and Kidney Diseases, The National Institutes of Health, Bethesda, MD, United States; ^2^ Department of Biological Sciences, Florida International University, Miami, FL, United States

**Keywords:** receptors, adenylate cyclases, protein kinases, protein phosphatases, phosphodiesterases, G proteins, ras, mTORC2

## Abstract

Self-organized and excitable signaling activities play important roles in a wide range of cellular functions in eukaryotic and prokaryotic cells. Cells require signaling networks to communicate amongst themselves, but also for response to environmental cues. Such signals involve complex spatial and temporal loops that may propagate as oscillations or waves. When *Dictyostelium* become starved for nutrients, cells within a localized space begin to secrete cAMP. Starved cells also become chemotactic to cAMP. cAMP signals propagate as outwardly moving waves that oscillate at ∼6 min intervals, which creates a focused territorial region for centralized cell aggregation. Proximal cells move inwardly toward the cAMP source and relay cAMP outwardly to recruit additional cells. To ensure directed inward movement and outward cAMP relay, cells go through adapted and de-adapted states for both cAMP synthesis/degradation and for directional cell movement. Although many immediate components that regulate cAMP signaling (including receptors, G proteins, an adenylyl cyclase, phosphodiesterases, and protein kinases) are known, others are only inferred. Here, using biochemical experiments coupled with gene inactivation studies, we model an integrated large, multi-component kinetic pathway involving activation, inactivation (adaptation), re-activation (re-sensitization), feed-forward, and feed-back controls to generate developmental cAMP oscillations.

## 1 Introduction

*Dictyostelium* exhibit one of the best-known bio-oscillators. During early development on solid substrata, *Dictyostelium* organize territorial waves of the chemoattractant extracellular cAMP that oscillate at ∼6 min intervals (Martiel and Goldbeter, 1985; Maeda et al., 2004; McMains et al., 2008; Kuhn et al., 2021). In the wild, single-cell *Dictyostelium* feed on bacterial sources. When nutrients within a territory become depleted, the starved *Dictyostelium* initiate a multi-cell developmental program. Cells begin to synthesize and secrete cAMP, which serves as a chemoattractant. Cells migrate toward the cAMP and relay the signal. Eventually, cells within a collected focal area form multicellular aggregates.

Secreted cAMP radiates as a wave, with peak/valley levels that vary by ∼1000-fold with a defined temporal periodicity. As a cAMP wave passes through a territory, cells oscillate through responsive, adapted, and de-adapted states for both chemotaxis and signal-relay. This ensures a unidirectional flow of the cAMP. Mechanistically, cAMP oscillations require 3 broad, group-step responses (McMains et al., 2008; Kuhn et al., 2021).

First, the enzyme for cAMP synthesis [adenylyl cyclase A (ACA)] must be activated and a primary intracellular phosphodiesterase (RegA) that degrades cAMP must be inactivated (Pitt et al., 1992; Shaulsky et al., 1998; Thomason et al., 1999; Kimmel and Parent, 2003; Manahan et al., 2004; Bader et al., 2007; Krishnamurthy et al., 2015; Kuburich et al., 2019; Kuhn et al., 2021). Collectively, these regulations ensure rapid intracellular and extracellular (secreted) high accumulation of cAMP from a low basal level. Secreted cAMP levels are regulated by the activity of the extracellular phosphodiesterase PDE1 (Franke et al., 1991; Garcia et al., 2009; Kriebel and Parent, 2009; Kawabe and Schaap, 2022).

To return cAMP to basal levels, the changed activities of both ACA and RegA must be reversed within minutes. ACA becomes inactivated and RegA is re-activated, and accumulated cAMP degraded. To ensure co-operative, continuous, and directionally outward relay of cAMP, both ACA and RegA activities must remain uncoupled from re-regulation, and so remain locked in their basal state in a set-time delay, with each cAMP oscillatory event initiating from the original focal region.

Finally, the pathways must be actively re-set to allow response to the next cAMP oscillatory signal.

**FIGURE 1:**
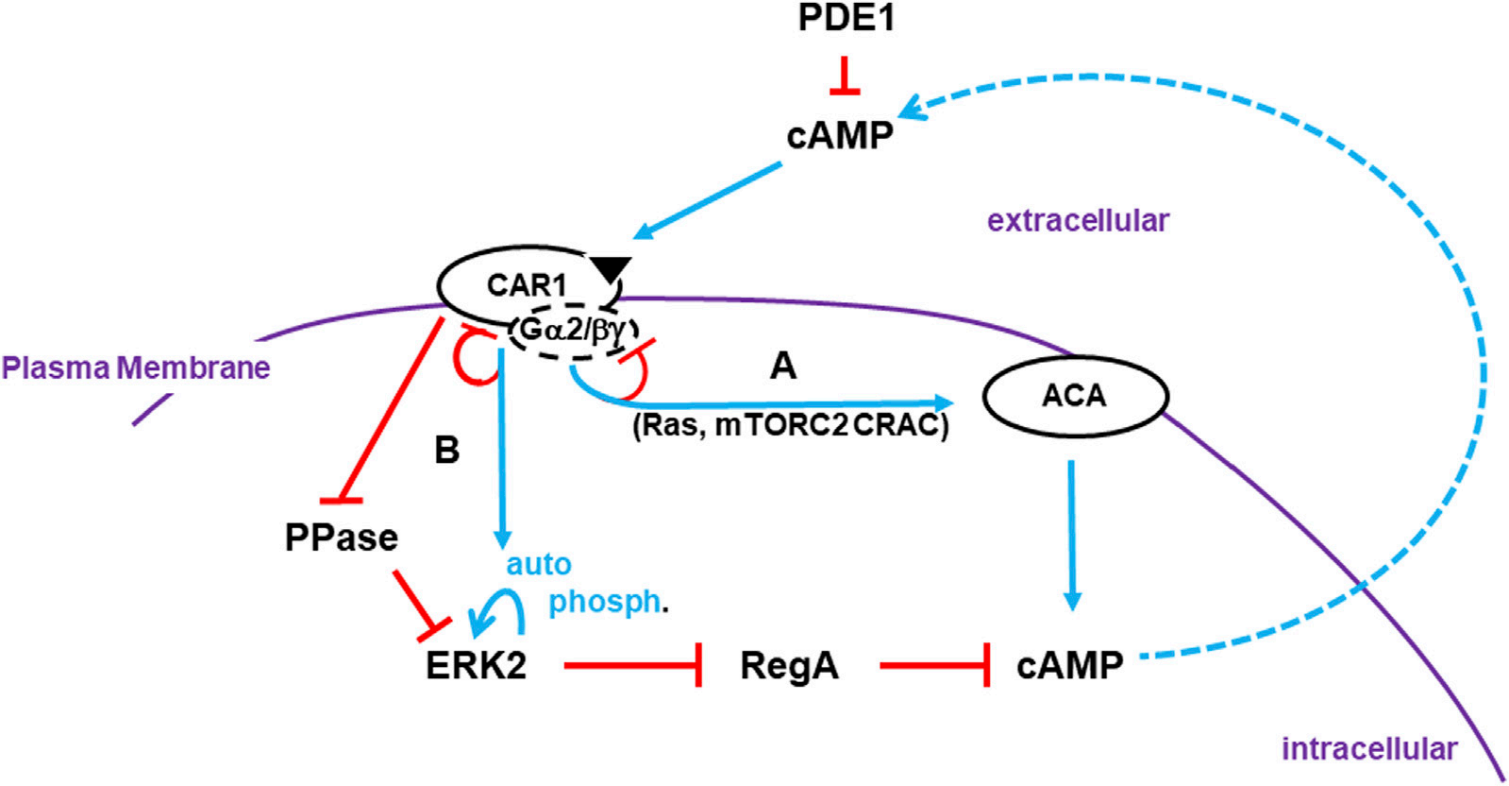
Activation of cAMP synthesis/accumulation. *Receptor Activation*: The cAMP (CAR1) receptors are surface chemoattractant GPCRs coupled to Gα2 and βγ subunits. Blue lines represent activation pathways, whereas red lines are inhibitory. Once stimulated, CAR1, bound to cAMP, activates a series of G protein-dependent (*e*.*g*., Ras, mTORC2) and G protein-independent (*e*.*g*., CAR1 phosphorylation) downstream effectors. **(A)** G protein-dependent circuits activate ACA (adenylyl cyclase A) for cAMP production. G protein-dependent signaling adapts. Receptors become de-sensitized to continuous, non-varying ligand stimulation and ACA signaling terminates. **(B)** ERK2 is activated by two pathways that are largely G protein-independent. ERK2 phosphorylation is auto-catalytic but is activated downstream of CAR1. The phosphatase (PPase) that acts on pERK2 is inhibited by activated CAR1. Although ERK2 kinase activity will adapt in the presence of saturating ligand concentrations, the PPase remains inactive and so pERK2 is persistent. *pERK2/RegA/cAMP*: Intracellular cAMP is degraded by RegA, an intracellular phosphodiesterase (PDE). pERK2 inhibits RegA, allowing accumulation of newly synthesized cAMP. Intracellular cAMP is secreted, where it can activate CAR1 and recruit other cells in cAMP relay. RegA has a putative docking site for ERK2 (Adhikari et al., 2020). While the docking site may facilitate RegA/ERK2 interactions, it is not essential. Extracellular cAMP is degraded by secreted PDE1 and CAR1 is inactive. Thus, pERK2 kinase is de-activated and RegA is re-activated, which degrades intracellular cAMP. *Developmental cAMP Oscillations*: During *Dictyostelium* development, oscillating waves of cAMP organize cells for multi-cellular aggregation. Cells go through cyclic co-ordinated rounds for activation/de-activation of ACA and de-activation/re-activation of RegA. Below we elaborate to the mechanistic pathways for these activations/de-activations in an integrated model (see Figure 4D, 5C, 6B; 7).

Many elements of the first phase have been described (see Figure 1). Activation of the G protein coupled receptor CAR1 induces the dissociation of the bound Gα2 and βγ subunits (Janetopoulos et al., 2001; Xu et al., 2010; Tariqul Islam et al., 2018; Adhikari et al., 2021). Released βγ subunits stimulate Ras, mTORC2, and other pathways, which are collectively required to activate ACA (Insall et al., 1994a; Lee et al., 2005; McMains et al., 2008; Cai et al., 2010; Liao et al., 2010; Liao et al., 2013; Jaiswal et al., 2019). Significantly, the ligand for CAR1 is cAMP itself. So, an initial limited activation of CAR1 promotes the continuous accumulation of cAMP, *via* the amplifying activation of ACA and inhibition of RegA (see Figure 1). That is, until cAMP levels rise sufficiently to saturate all CAR1 binding sites. At this point, ligand saturation of CAR1 leads to receptor pathway inhibition/adaptation (desensitization), and the de-activation of ACA (see Figure 1). Thus, ACA activators Ras and mTORC2 also oscillate with the same timing of cAMP, but with activity peaks that temporally precede full cAMP accumulation (Insall et al., 1994a; Lee et al., 2005; McMains et al., 2008; Cai et al., 2010; Liao et al., 2010; Liao et al., 2013; Jaiswal et al., 2019).

Regulation of RegA, an intracellular cAMP PDE, is known to be inhibited by phospho-activated ERK2 [pERK2 (Shaulsky et al., 1998; Maeda et al., 2004)]. Yet, ERK2 regulation is complex and has not been fully integrated mechanistically within a model for cAMP oscillation control (Segall et al., 1995; Maeda et al., 2004; Brzostowski and Kimmel, 2006; Brzostowski et al., 2013). CAR1 stimulation drives the phosphorylation and activation of ERK2 (Hereld et al., 1994; Brzostowski and Kimmel, 2006; Cao et al., 2014) and, although CAR1 is G protein coupled, Gα and βγ subunits do not play a significant role for pERK2 (Maeda et al., 1996; Brzostowski and Kimmel, 2006) (see Figure 1). Further, two separate modes downstream of CAR1 are required for pERK2 activation; one promotes phosphorylation and the other inhibits de-phosphorylation (Brzostowski and Kimmel, 2006; Brzostowski et al., 2013). ERK2 phosphorylation follows standard kinetics for receptor adaptation, whereas phosphatase (PPase) inhibition of ERK2 is non-adaptive (Brzostowski and Kimmel, 2006).

*Dictyostelium* ERK2 is a member of broad class of Extracellular-Regulated/Mitogen-Activated protein kinases (ERKs/MAPKs), encompassing subfamilies ERK1/2, JNK, p38, and many others, that are highly conserved from yeasts to mammals (Kim and Choi, 2010). In many systems, MAPKs/ERKs function as central regulators of multiple pathways, including cell growth, migration, and differentiation. Classically, MAPKs/ERKs receive activation signals from surface receptors responsive to extracellular environmental cues. These signals are integrated through multi-protein kinase cascades (Kim and Choi, 2010; Lavoie et al., 2020). Cell-surface receptors mediate activation of MEK Kinase Kinases (*e*.*g*., Raf, MAPKKK), which phosphorylate/activate dual-specific MEKs (MAPK/ERK Kinases, MAPKK), which phosphorylate/activate ERKs/MAPKs, at a conserved pTEpY motif (Lavoie et al., 2020; Barbosa et al., 2021). It is now clear, however, that ERK/MAPK regulatory circuits are far more complex, involving more factor inputs. Certain ERKs, including *Dictyostelium* ERK2, do not require MEK, but have inherent capacity for auto-phosphorylation (Nichols et al., 2019). Comparative sequence analyses of the *Dictyostelium* protein kinome have identified only 2 ERKs (ERK1 and ERK2), a single MEK (MEK1), and a single MEKK (MEKKα) (Kimmel, 2005; Mendoza et al., 2005; Goldberg et al., 2006). Although ERK regulations in *Dictyostelium* have differences to many mammalian type pathways, null mutation analyses place ERKs as essential to chemotactic gradient formation and sensing (Gaskins et al., 1996; Brzostowski and Kimmel, 2006; Nguyen and Hadwiger, 2009; Hadwiger and Nguyen, 2011; Brzostowski et al., 2013; Schwebs and Hadwiger, 2015; Schwebs et al., 2018; Nichols et al., 2019; Hadwiger et al., 2022).

Several circuitry models have been proposed to explain the generation of cAMP oscillations, however, many elements as assembled do not function as originally modeled. Here, incorporating new data, we propose an extended model that directly integrates more than 20 components, and invokes as many others, in feed-forward and feed-back inhibitions/activations for a pathway logic that describes a mechanism to direct the observed cAMP oscillations during early *Dictyostelium* development that oscillate 1000-fold with a repeating, temporal cycle.

## 2 Results

### 2.1 pERK2 and pERK1 oscillate during cAMP chemotactic developmental signal-response

**FIGURE 2:**
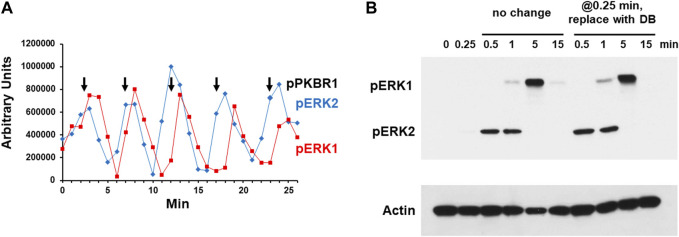
Chemoattractant stimulated phosphorylation of ERK1 and ERK2. **(A)** Spontaneous oscillations of ERK1 and ERK2 phosphorylation. Cells were pulsed in developmental buffer (DB) to a final concentration of 75 nM cAMP every 6 min for 5 h. Cells were washed, resuspended in fresh buffer, and incubated for 30 min without exogenous cAMP to allow spontaneous cAMP oscillations. Aliquots were collected at 1-min intervals and ERK1 and ERK2 phosphorylations assayed by immunoblot. For pERK1 and pERK2 we used an antibody specific to their phospho-forms. Shown are the time courses of quantified relative signal intensities (see Supplementary Figure S2). In parallel, we assayed PKBR1 phosphorylation at pT470, which also oscillates (Liao et al., 2013); peak value positions are shown as reference. **(B)** cAMP-stimulated phosphorylation of ERK1 and ERK2. Cells in DB were plated in microtiter dishes and stimulated with 10 μM cAMP at time 0. One set of wells was left unchanged and samples were taken at times indicated. For another set of wells, the cAMP was removed at 0.25 min (15 s) and replaced with DB, and samples were taken at times indicated. ERK1 and ERK2 phosphorylations and actin levels were assayed by immunoblot.

During early development on solid substrata, *Dictyostelium* organize territorial waves of extracellular cAMP, with ∼6 min oscillations (McMains et al., 2008; Kuhn et al., 2021). These oscillations are recapitulated in shaking cultures of developing *Dictyostelium*, where extracellular cAMP is secreted, accumulated, and degraded to 5’-AMP, also with ∼6-minute repeating cycles [Supplementary Figure S1 (Kimmel, 1987; McMains et al., 2008)]. Biochemical studies show that kinases AKT and PKBR1 are phosphorylated within ∼15 s of stimulation by exogenous cAMP, followed by pERK2 at ∼30 s, and we and others have shown that the measured temporal changes in extracellular cAMP are reflected in kinase pathway activations, downstream of cAMP receptor CAR1 (Brzostowski and Kimmel, 2006; Hadwiger and Nguyen, 2011; Brzostowski et al., 2013; Liao et al., 2013). Thus, in shaking culture phospho-activations of kinases AKT, PKBR1, and ERK2 also oscillate at 6 min intervals, with pERK2 slightly delayed relative to pAKT/pPKBR1 (Liao et al., 2013). During the course of such studies, we observe that pERK1 also oscillates with identical kinetics, but slightly delayed, relative to pERK2 (Figure 2A and Supplementary Figure S2).

Others have observed that cells stimulated in culture with cAMP will activate pERK1 in delay compared to that of pERK2 and suggested that pERK1 may involve pERK2 (Hadwiger and Nguyen, 2011; Schwebs and Hadwiger, 2015; Schwebs et al., 2018; Kimmel, 2019; Nichols et al., 2019). We wished to confirm this observation and understand the connection more mechanistically. We stimulated cells with 10 μM cAMP, and although extracellular cAMP is degraded with time, a 10 μM stimulus ensures that cAMP remains elevated through >3 min (Brzostowski and Kimmel, 2006). We show maximal pERK2 at 0.5–1 min, which then rapidly declines and remains suppressed through 15 min (Figure 2B and Supplementary Figure S3). pERK1 appears with a definitive lag relative to pERK2; then pERK1 also declines. Identical data are obtained in the absence of developmental dependency (see below), which we had previously shown to be fully sensitive to activation by cAMP for cAMP production, Ca^+2^ influx, Ras-GTP, pAKT, pPKBR1, and pERK2 (Liao et al., 2013).

To determine if a single dose of cAMP were sufficient to elicit these precise responses, we stimulated cells with 10 μM cAMP for 15 s, but then completely removed the stimulus, replacing cAMP with fresh DB. Identical sample times were examined as the controls. As seen, the pERK2 and pERK1 responses were identical in both cultures (Figure 2B). A single cAMP dose was sufficient to induce pERK2 activation/inactivation, followed by pERK1 activation/inactivation, essentially mimicking a single oscillation wave. Since the exogenously added cAMP was present through only 15 s, the data make it likely that ERK1 is activated and deactivated as part of a downstream cascade circuit, rather than directly *via* CAR1. Potentially pERK2 may be required to activate ERK1, and further, since pERK2 decline seems to follow pERK1 activation (Figure 2A), pERK1 may be required to suppress pERK2.

### 2.2 pERK2 induces pERK1

**FIGURE 3:**
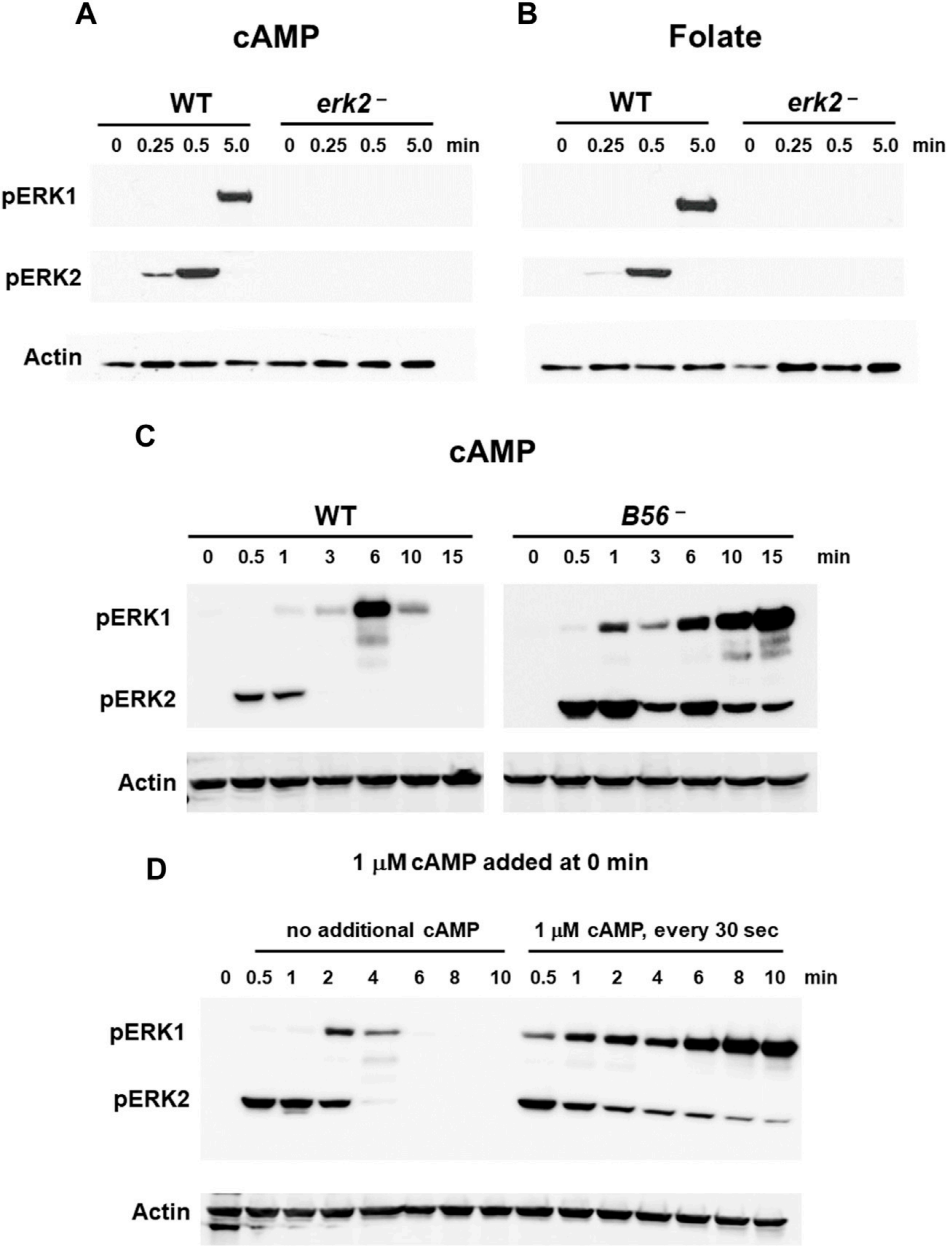
ERK1 activation is regulated by pERK2. **(A)** cAMP-stimulated phosphorylation of ERK1 requires ERK2 activation. WT and *erk2*-null cells were pulsed to a final concentration of 75 nM cAMP every 6 min for 5 h, and then stimulated with 10 μM cAMP at time 0. Aliquots were collected at indicated times and ERK1 and ERK2 phosphorylations and actin levels assayed by immunoblot. **(B)** Folate-stimulated phosphorylation of ERK1 requires ERK2 activation. WT and *erk2*-null cells were shaken in DB culture for 90 min and stimulated with 1 μM folate. Aliquots were collected at indicated times and ERK1 and ERK2 phosphorylations and actin levels assayed by immunoblot. **(C)** pERK1 is extended in cells lacking the PPase for pERK2. WT and *B56*-null cells were pulsed in developmental buffer (DB) to a final concentration of 75 nM cAMP every 6 min for 5 h, and then stimulated with 10 μM cAMP at time 0. Aliquots were collected at indicated times and ERK1 and ERK2 phosphorylations and actin levels assayed by immunoblot. **(D)** Inhibition of pERK2 de-phosphorylation extends pERK1. WT cells were pulsed in developmental buffer (DB) to a final concentration of 75 nM cAMP every 6 min for 5 h, and then stimulated with 10 μM cAMP at time 0. One set of cells was left untreated. Another set was further stimulated with 1 μM cAMP every 30 s. Aliquots were collected at indicated times and ERK1 and ERK2 phosphorylations and actin levels assayed by immunoblot.

We directly examined the activation of ERK1 in WT cells and cells lacking the *ERK2* gene. As previously suggested (Hadwiger and Nguyen, 2011; Schwebs and Hadwiger, 2015; Schwebs et al., 2018; Kimmel, 2019; Nichols et al., 2019), *erk2*-null cells are unable to activate pERK1 following stimulation with cAMP (Figure 3A), data consistent with a dependent role for pERK2 in ERK1 regulation. To ensure that the phenotype was not secondary to a developmental defect or even a cAMP response, we additionally looked to ERK2/ERK1 regulation in growth-phase cells that are responsive through a different chemoattractant, folate, and a different GPCR, Far1 (Figure 3B). We observe fundamentally similar time course regulations for ERK2 and ERK1 in control cells, and again with full dependency on pERK2 for ERK1 phosphorylation. We then expressed a FLAG-ERK2 fusion in *erk2*-null cells (Supplementary Figure S4) to further follow pERK1 dependency on pERK2. These cells show rescued cAMP-dependent activation of pERK2 and rescued activation of pERK1, which were temporally similar to WT (Supplementary Figure S4).

If pERK2 were required for the regulation of ERK1 activation, we hypothesized that maintaining persistent pERK2 levels might similarly extend pERK1 activation. We approached this in two manners.

Two phosphatases (PPases) are involved in ERK2 de-phosphorylation during early development, Protein Phosphatase 2A (PP2A) and Mpl1 (Rodriguez et al., 2008), with PP2A the more significant (Rodriguez et al. al., 2008; (Lee et al., 2008); data not shown). We, thus, focused to the regulation of pERK2 and pERK1 comparing WT to *B56-*null cells, which lack the essential B56 regulatory subunit of PP2A (Lee et al., 2008). In *B56*-null cells, ERK2 is hyper-phosphorylated and pERK2 levels are significantly extended temporally compared to WT (Figure 3C). Further, ERK1 remains phosphorylated through the entire time course, consistent with phosphorylation of ERK1 induced through pERK2. Fundamentally similar data are obtained in growing cells, using folate as the stimulus (Supplementary Figure S5A).

Next, we maintained pERK2 levels through inhibition of ERK2 de-phosphorylation (Brzostowski and Kimmel, 2006). We had previously shown that kinase and PPase activities for ERK2 were under separate regulations. The kinase is transiently activated (see Figure 1) but is then rapidly adapted to continuous cAMP stimulation. Conversely, inhibition of the PPase for ERK2 persists in the presence of continuous receptor stimulation. Here, we added 1 μM cAMP every 30 s to maintain CAR1 saturation and PPase inhibition. As seen (Figure 3D), inhibition of PPase with continued cAMP stimulation suppresses pERK2 de-phosphorylation; persistent pERK2 activation is followed with prolonged pERK1 activation. The kinetics of both pERK2 and pERK1 are extended compared to controls that had received only single cAMP dose (Figure 3D), again supporting activation of pERK1 downstream of pERK2.

We performed a parallel experiment in growing cells, using folate as the stimulus. When 1 μM folate is added every min to maintain Far1 receptor saturation, inhibition of PPase is also persistent, limiting de-phosphorylation of ERK2. Continuous pERK2 activation promotes continued pERK1 activation. Again, the kinetics of both pERK2 and pERK1 are extended compared to controls that had received only a single folate dose (see Supplementary Figure S5B).

Persistent extracellular cAMP levels can also be generated through direct inhibition of the extracellular phosphodiesterase PDE1 with 10 mM DTT (Brzostowski and Kimmel, 2006). Maintenance of a saturating extracellular cAMP stimulus with 10 mM DTT also inhibits de-phosphorylation of pERK2, which supports persistent pERK1 activation. We show that both pERK2 and pERK1 are extended in DTT-treated cells, compared to untreated cells (Supplementary Figure S5C).

### 2.3 MEK1/ERK1 feed-back suppresses pERK2

**FIGURE 4:**
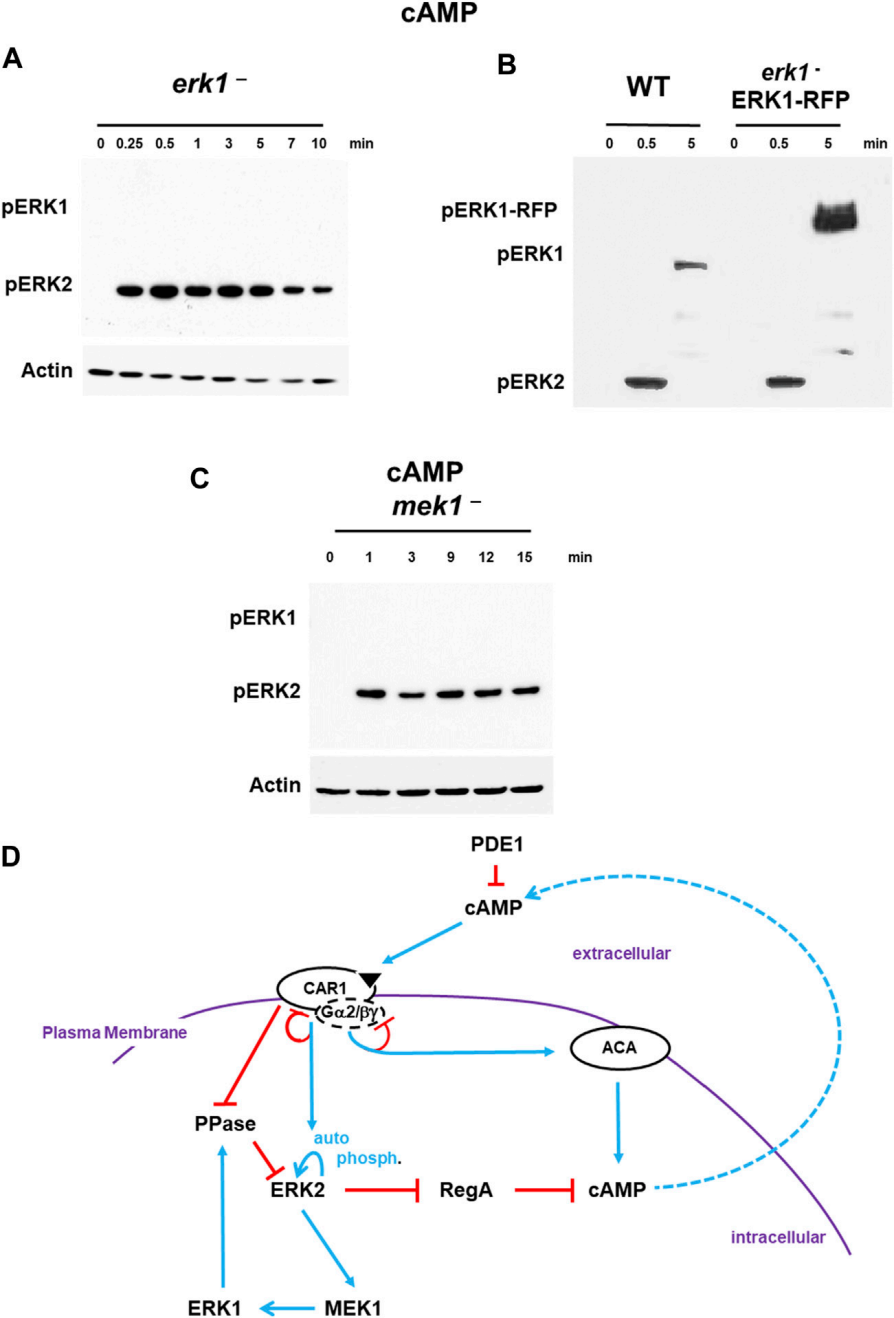
De-phosphorylation of pERK2 is regulated by MEK1/ERK1. **(A)**. De-phosphorylation of pERK2 is regulated by ERK1 activity. WT and *erk1*-null cells were pulsed to a final concentration of 75 nM cAMP every 6 min for 5 h, and then stimulated with 10 μM cAMP at time 0. Aliquots were collected at indicated times and ERK1 and ERK2 phosphorylations and actin levels assayed by immunoblot. **(B)** ERK1-RFP rescues de-phosphorylation of pERK2 in *erk1*-null cells. An ERK1-RFP fusion was expressed in *erk1*-null cells. WT and *erk1*-null cells expressing ERK1-RFP (*erk1* ^-^/ERK1-RFP) were pulsed to a final concentration of 75 nM cAMP every 6 min for 5 h, and then stimulated with 10 μM cAMP at time 0. Aliquots were collected at indicated times and ERK1 and ERK2 phosphorylations and actin levels assayed by immunoblot. The MW of ERK1 is ∼60 kDa. The MW of ERK1-RFP is ∼90 kDa. **(C)** MEK1 is required for pERK1 and de-phosphorylation of pERK2. WT and *mek1*-null cells were pulsed to a final concentration of 75 nM cAMP every 6 min for 5 h, and then stimulated with 10 μM cAMP at time 0. Aliquots were collected at indicated times and ERK1 and ERK2 phosphorylations and actin levels assayed by immunoblot. **(D)** pERK2 regulates pERK1 and pERK1 regulates de-phosphorylation of pERK2. The model for activation of cAMP synthesis/accumulation (see Figure 1) is extended; blue lines represent activation pathways, whereas red lines are inhibitory. pERK2 is required for MEK1-mediated activation of pERK1, although data do not suggest MEK1 as a phospho-target of ERK2 (Nichols et al., 2019). pERK1 leads to de-activation of pERK2, the reactivation of RegA, and the degradation of intracellular cAMP.

With regulation of ERK1 defined downstream of ERK2, we sought to re-visit the temporal connection between ERK1 activation and ERK2 de-phosphorylation. We hypothesized that in the absence pERK1, ERK2 should not exhibit downregulation but shown substantially persistent phosphorylation. Indeed, cells deficient for ERK1, show activation of pERK2, without a rapid de-phosphorylation response to either cAMP (Figure 4A) or folate (Supplementary Figure S6).

Next, we expressed an ERK1-RFP fusion in *erk1*-null cells. These cells show rescued activation of pERK1 at a mobility consistent with the size of the ERK1-RFP fusion, and also pERK2 activation and rescued de-phosphorylation of pERK2 that were temporally similar to WT (Figure 4B).

Although ERK2 is argued to have characteristics similar to other ERKs that are not phosphorylated by MEKs, previous work in *Dictyostelium* has suggested MEK1 as the pERK1 activator (Ma et al., 1997; Sobko et al., 2002; Mendoza et al., 2005; Nguyen et al., 2010). We confirm these studies and further show that *mek1*-null cells, which lack pERK1, also show extended pERK2, paralleling the previous studies using *erk1*-nulls (Figure 4C). Expression of a GFP-MEK1 fusion in *mek1*-null cells (Supplementary Figure S7) showed rescued cAMP-dependent activation of pERK1 but also rescued de-phosphorylation of pERK2, which temporally parallels WT (Supplementary Figure S7).

Thus, following CAR1 activation, we observe a rapid activation of pERK2, leading to MEK1-mediated phosphorylation of pERK1, which, we suggest, feed-back inhibits pERK2, probably through activation of a PPase (Figure 4D). With de-phosphorylation of pERK2, RegA is reactivated, bringing the elevated levels of cAMP back to base line, which defines a single cAMP oscillation.

### 2.4 cAMP/PKA suppresses pERK1

As stated previously, a major known mechanistic action of pERK2 is in control of intra- and extra-cellular levels of cAMP (Sawai et al., 2005; Brzostowski and Kimmel, 2006), where it functions to inhibit RegA, the major intracellular PDE that degrades cAMP (Sawai et al., 2005). Thus, the co-activation of ACA and pERK2 by extracellular cAMP, leads to intracellular cAMP accumulation and activation of the cAMP-dependent protein kinase (PKA). Mechanistically, this occurs through cAMP binding to the regulatory subunit of PKA (PKAreg), which promotes dissociation from and activation of the PKA catalytic subunit (PKAcat). Several models had invoked PKAcat to feedback inhibit ACA or ERK2 directly (Maeda et al., 2004), although subsequent published data do not support these conclusions (Sawai et al., 2005; Brzostowski and Kimmel, 2006). Still, possibly PKAcat might mediate pERK2-induced activation of ERK1. For this, we studied cells with diminished cAMP/PKA signaling [*acaA*-nulls (Hirose et al., 2021)] or with constitutively active PKA [*pkaR*-nulls (Shaulsky et al., 1998)].

**FIGURE 5:**
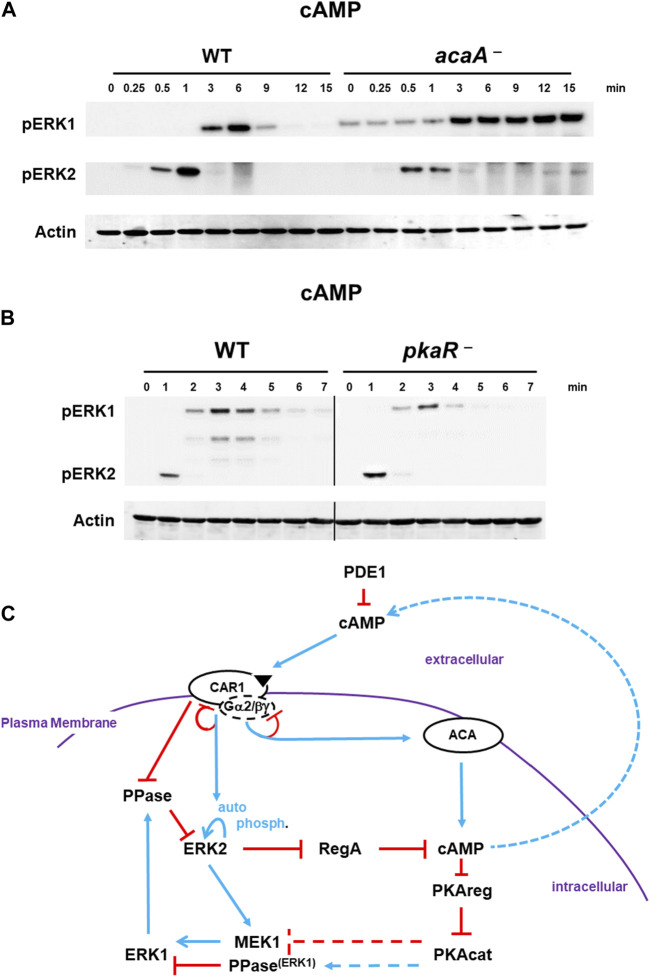
De-phosphorylation of pERK1 is regulated by cAMP/PKA signaling. **(A)**. Diminished cAMP/PKA signaling inhibits de-phosphorylation of pERK1. WT and *acaA*-null cells were pulsed to a final concentration of 75 nM cAMP every 6 min for 5 h, and then stimulated with 10 μM cAMP at time 0. Aliquots were collected at indicated times and ERK1 and ERK2 phosphorylations and actin levels assayed by immunoblot. **(B)** Constitutively active PKA enhances pERK1 de-phosphorylation. WT and *pkaR*-null cells were pulsed to a final concentration of 75 nM cAMP every 6 min for 5 h, and then stimulated with 200 nM cAMP at time 0. Aliquots were collected at indicated times and ERK1 and ERK2 phosphorylations and actin levels assayed by immunoblot. **(C)** cAMP/PKA feedback inhibits pERK1. The model for activation of cAMP synthesis/accumulation (see Figure 1) coupled to re-activation of cAMP degradation is extended (see Figure 4D; blue lines represent activation pathways, whereas red lines are inhibitory. cAMP-activated PKA promotes inactivation of pERK1, allowing re-activation of pERK2 upon a new signal/response. Cells lacking ACA have low PKA activity and extended pERK1; cells lacking PKAreg have constitutively active PKA and enhanced pERK1 de-phosphorylation. At present, we are unable to determine if activated PKA reduces MEK1 activity or if activated PKA promotes a phosphatase.

Interestingly, pERK2 and pERK1 are activated in *acaA*-null cells with similar kinetics to WT (Figure 5A). Thus, a pERK2-dependent increase in cAMP/PKA is not required to drive ERK1 activation. However, pERK1 in cells lacking ACA (*acaA* ^−^ cells) is significantly extended relative to WT (Figure 5A), regardless that ERK2 is de-activated. *Dictyostelium* possess two adenylate cyclases, ACR and ACG, in addition to ACA. These other ACs do not couple with CAR1, but are regulated differently (Hirose et al., 2021). We, thus, followed pERK2/pERK1 regulation in *acaA–/acrA*–*/acgA*–null cells (Hirose et al., 2021), which lack all 3 adenylate cyclases and fail to produce any detectable cAMP (Hirose et al., 2021). Results were identical to that of *acaA*-null cells (Supplementary Figure S8A). In the absence of cAMP and activated PKA, pERK1 remains elevated through the entire time course. We observed a similar loss of pERK1 de-activation in folate-stimulated cells that lack either ACA or PKAcat (Supplementary Figure S8B).

Based on data above (Figure 5A, Supplementary Figure S8), where reduced cAMP leads to extended pERK1, cells with constitutively activated PKA might be expected to show reduced pERK1 signaling than WT. To this, we used cells lacking the inhibitory regulatory subunit of PKA, *pkaR*-null cells (Shaulsky et al., 1998). Indeed, pERK1 activation is attenuated and de-phosphorylation more rapid in *pkaR*-null with constitutively elevated PKA than in WT (Figure 5B).

Collectively, we suggest PKA activation directs the de-phosphorylating, inactivation loop to pERK1 (Figure 5C). We are unable to determine if PKA suppresses MEK1 activity or if PKA promotes pERK1 de-phosphorylation. Regardless, the low basal level of pERK1 observed in both *acaA*-null and *acaA–/acrA*–*/acgA*–null cells, which have suppressed PKA activity, is consistent with PKA-regulated reduction in pERK1 levels.

### 2.5 ACA de-activation through receptor adaptation

Developmental cAMP oscillations are characterized by a rise in cAMP synthesis and accumulation, followed by cessation of cAMP synthesis and cAMP degradation, with the cycle repeating at defined temporal cycles. GPCR de-sensitization to a saturating or a continuous stimulation is a well-described phenomenon, often involving homologous (or heterologous) ligand-induced receptor phosphorylation (Xiao et al., 1999; Artemenko et al., 2016). *Dictyostelium* CAR1 is also modified by cAMP-induced phosphorylation (Klein et al., 1985; Hereld et al., 1994) and, compared to WT, CAR1 mutants with reduced phosphorylation show extended ACA activity in response to a persistent cAMP stimulation (Brzostowski et al., 2013). There are components downstream of CAR1 [*e*.*g*., RAS inhibition through GAP activation (McMains et al., 2008; King and Insall, 2009; Charest et al., 2010; Swaney et al., 2010; Rappel and Firtel, 2012; Liao et al., 2013; Scavello et al., 2017)] that also function to suppress ACA, and others have suggested that these pathways are the more significant for ACA suppression (Cai et al., 2010; Scavello et al., 2017). However, we have shown that cells that are fully insensitive to cAMP stimulation (*e*.*g*., RAS, mTORC2 activation) are also fully responsive to activation through a different GPCR, Far1 (Liao et al., 2013; Meena and Kimmel, 2017). Therefore, we have argued that primary adaptation is localized high in the circuit involving CAR1 and its associated G proteins and that downstream inhibitory feedback are more transitory.

**FIGURE 6:**
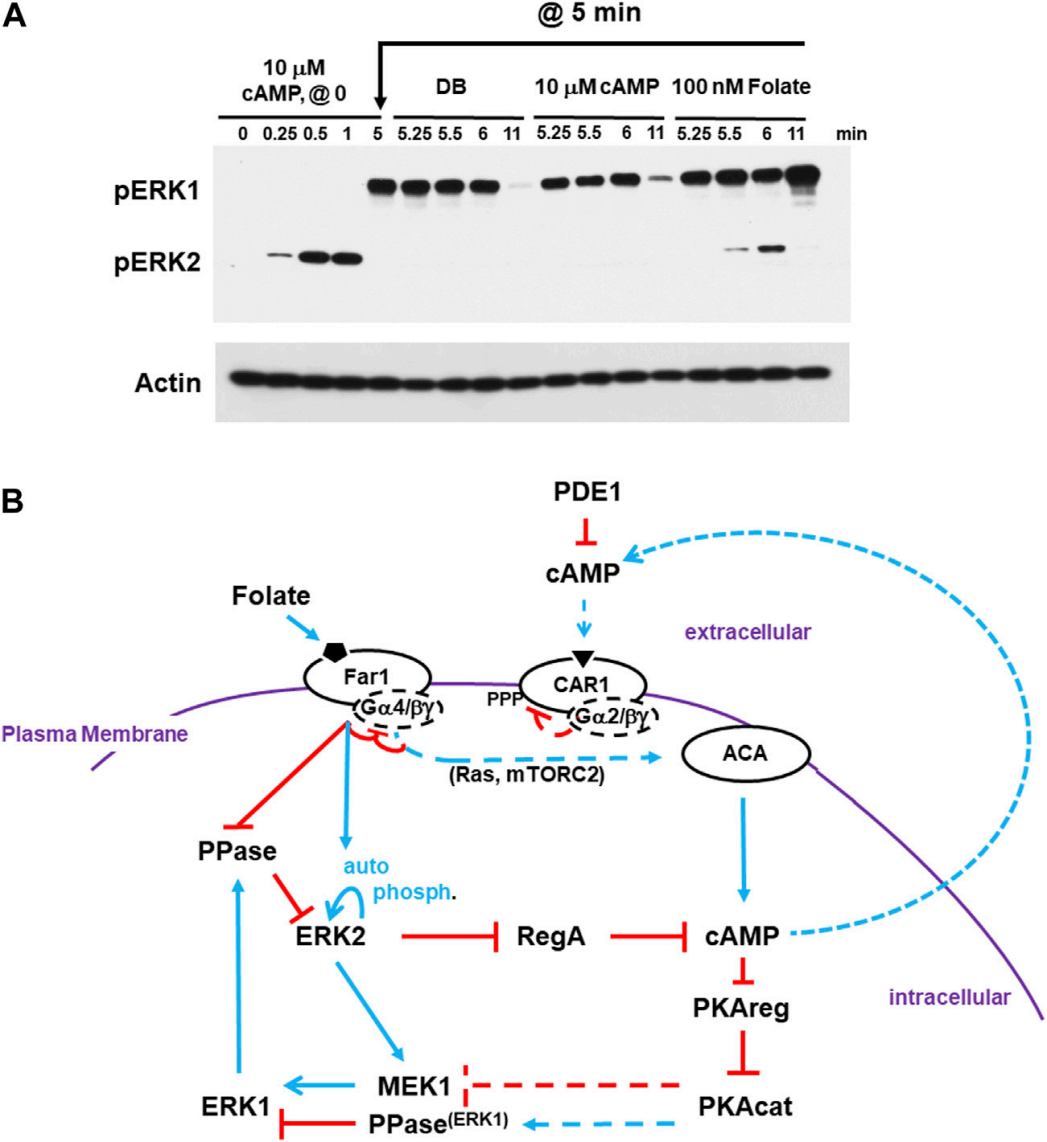
cAMP-adapted cells are fully responsive to folate stimulation. **(A)** cAMP-adapted cells are fully responsive to folate stimulation. Cells in DB were plated in microtiter dishes and stimulated with 10 μM cAMP at time 0. One set of cultures was given a small aliquot of DB at 5 min. One set of cultures was given an additional dose of 10 μM cAMP in DB at 5 min. One set of cultures was given a dose of 100 nM folate in DB at 5 min. Aliquots were collected at indicated times and ERK1 and ERK2 phosphorylations and actin levels assayed by immunoblot. **(B)** Cells desensitized to cAMP do not exhibit receptor cross-adaptation and are fully responsive to folate stimulation. The model for activation/de-activation of cAMP synthesis/accumulation (see Figures 1, 4D, 5C) is extended; blue lines represent activation pathways, whereas red lines are inhibitory. Cells that are CAR1 de-sensitized, and so unresponsive, to cAMP remain fully responsive to stimulation of folate receptor Far1. ACA adaptation to cAMP is primarily controlled proximal to CAR1. Upon CAR1 activation, G proteins dissociate, CAR1 is phosphorylated, and binding affinity to cAMP is reduced. While CAR1 is still able to engage cAMP, CAR1 does not signal-transmit. Although pathways more downstream may be inhibited (*e*.*g*., RAS suppression by GAP), these inhibitions are transient and by-passed by Far1 signaling. Like extracellular cAMP, extracellular folate is subject to enzymatic inactivation. For folate, this involves de-amination at position 2.

Thus, we explored the regulation of ERK2, and, accordingly RegA and cAMP, in response to differential GPCR ligand-induced stimulation. First, cells were stimulated with saturating 10 μM cAMP. We observed rapid activation of pERK2, followed by ERK2 de-phosphorylation and activation of pERK1. At 5 min, CAR1 is still in a desensitized state (Liao et al., 2013). Here, the culture was split. An aliquot was washed of cAMP and replaced with buffer without cAMP (DB). A separate aliquot was washed of cAMP and replaced with fresh 10 μM cAMP. Both show identical regulatory patterns for ERK2 and ERK1. ERK2 remains inactive and pERK1 retains activity until later time points, when it also declines (Figure 6A). Additionally, we activated an additional aliquot at 5 min with folate. The GPCR folate receptor is coupled to Gα4, and not Gα2 as is CAR1, but the same Gβγ. In these cells, we see a new cycle of ERK2 response and de-phosphorylation and ERK1 re-activation. (Figure 6A). In a similar experiment, we had shown cAMP adapted cells were responsive to folate for Gβγ-dependent re-activation of RAS and mTORC2 in a path toward activated ACA (Liao et al., 2013; Meena and Kimmel, 2017). Therefore, the primary adaptation for ACA must be independent of downstream pathway feed-back.

To reinitiate signal propagation, receptors must become re-sensitized (de-adapted). De-adaptation is not fully understood but involves receptor de-phosphorylation and G protein re-coupling. The individual downstream inhibitory modules certainly play critical roles for acute aspects to cAMP signaling, but they are bypassed upon receptor stimulation. Therefore, we suggest that receptor adaptation/re-sensitization is the more critical to allow oscillatory response/relay. Once all the pathways are fully re-set, a slight activation of CAR1 is sufficient to initiate another cycle of cAMP amplification and oscillation (see Figures 1, 6B).

## 3 Discussion

### 3.1 A Co-ordinated, mechanistic model for cAMP oscillations

During early development of *Dictyostelium*, secreted cAMP serves as a chemoattractant to organize cells at centers of aggregation (Meima and Schaap, 1999; Jaiswal et al., 2012a; Jaiswal et al., 2012b; Fukujin et al., 2016; Scavello et al., 2017; Singer et al., 2019), The receptors for cAMP (CAR1) are surface chemoattractant GPCRs, coupled to Gα2 and Gβγ subunits, which transduce the extracellular signal for directed cell movement (Janetopoulos et al., 2001; Xu et al., 2010; Tariqul Islam et al., 2018; Adhikari et al., 2021). In addition, CAR1 orchestrates a periodic activation/de-activation/re-activation pathway for cAMP generation and clearing, which relays the cAMP signal (Louis et al., 1993; Insall et al., 1994b; Hereld et al., 1994; Thomason et al., 1999; McMains et al., 2008; Kriebel and Parent, 2009; Xu et al., 2010; Brzostowski et al., 2013; Cao et al., 2014). In three-dimensional space, cAMP is seen as waves, oscillating with a set period. The cAMP waves move outward from an initial center, with responsive cells migrating “up” the wave gradient and relaying the cAMP to co-ordinate more cells. To ensure inward directional movement and outward cAMP relay as the wave pass through a territory, cells go through activated and adapted states. In shaking culture, coordinated cAMP oscillations are seen through the entire culture as peaks and valleys with periodicities of ∼6 min (see Supplementary Figure S1).

**FIGURE 7:**
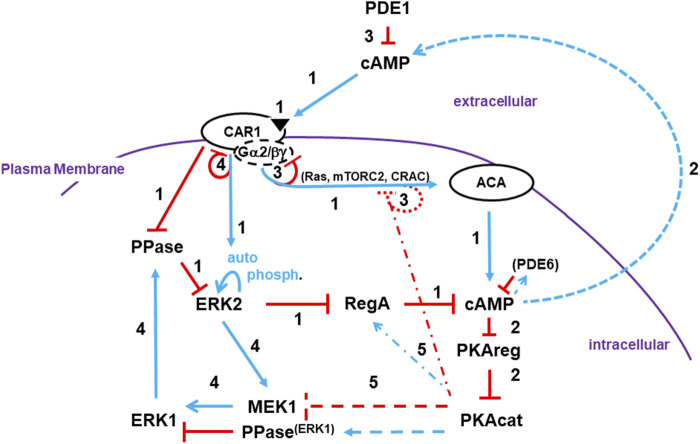
A pathway model involving activating/adaptive and feed-forward/feed-back loops for directed oscillatory cAMP signaling. The collective model for activation/de-activation of cAMP synthesis/accumulation (see Figures 1, 4D, 5C, 6B) is extended; blue lines represent activation pathways, whereas red lines are inhibitory. The circuit integrations describe a mechanistic sequence that generates cAMP oscillations through a series of temporally spaced activating and inhibitory actions. See text for more details. Step 1: cAMP synthesis/accumulation (see discussion at 3.1.1). Step 2: cAMP compartments (see discussion at 3.1.2). Step 3: De-activation of ACA (see discussion at 3.1.3). Step 4: Intracellular cAMP degradation (see discussion at 3.1.4). Step 5: Target modes toward a re-responsive basal state (see discussion at 3.1.5).

We present an integrated, cross-regulation pathway model involving activating/adaptive and feed-forward/feed-back loops, for directed oscillatory cAMP signal-relay/response during the development of *Dictyostelium* (Figure 7). Blue lines represent activation pathways, whereas red lines are inhibitory. In the model, we highlight the most critical elements for temporally regulated activating/inhibitory oscillating circuitry. Other components with significant input roles are discussed in more detail below. Still, we recognize that we have not included every cAMP signaling component, with some having possible feed-back/feed-forward modulating influences.

#### 3.1.1 cAMP synthesis/accumulation

##### 3.1.1.1 cAMP receptor CAR1 activation

Once stimulated by cAMP, CAR1 activates a series of common G protein-dependent (*e*.*g*., Ras, mTORC2, CRAC) and -independent (*e*.*g*., Ca^+2^ influx, GPCR phosphorylation) downstream effectors.

##### 3.1.1.2 ACA activation

CAR1/G protein-dependent circuits activate ACA (adenylyl cyclase A) for cAMP production. Multiple downstream pathways act collectively for ACA activation. In *Dictyostelium,* most ACA actions are dependent upon released Gβγ (Lee et al., 2005; McMains et al., 2008; Liao et al., 2013), rather than a Gα-GTP, as for most ACs in mammalian cells. However, Gα9 is a CAR1-coupled inhibitory subunit (Brzostowski et al., 2002).

There are many collective inputs to ACA downstream of CAR1. Examples include Ras proteins and their downstream effectors. RasG and RasC are small GTPases activated by GEFs (GTP exchange factors) and deactivated by GAPs (GTPase activating proteins) (Cai et al., 2010; Williams et al., 2019). Both GEFs and GAPs are suggested to be activated by CAR1, but with different temporal kinetics. GAP activity is delayed, so CAR1 promotes rapid formation of RasG-GTP and RasC-GTP complexes. (Scavello et al., 2017).

RasG-GTP activates PI3K and synthesis of membrane-bound PIP_3_, which recruits a series of binding proteins (Funamoto et al., 2002; Sasaki et al., 2007; Fukushima et al., 2019), including CRAC (Cytosolic Regulator of Adenylyl Cyclase), an essential regulator of ACA (Insall et al., 1994a). RasC-GTP is required to activate mTORC2 (Lee et al., 2005; McMains et al., 2008; Cai et al., 2010; Liao et al., 2010; Liao et al., 2013; Jaiswal et al., 2019), a multi-protein protein kinase with protein kinases AKT and PKBR1 as primary activating targets.

##### 3.1.1.3 ERK2 activation/RegA inhibition

RegA is a primary intracellular PDE for cAMP (Thomason et al., 1999; Adhikari et al., 2020). RegA is a response-regulator of a two-component phospho-relay system (Thomason et al., 1999). While the upstream sensor histidine kinase during early development is not identified, RdeA is a phospho-relay intermediate, where downstream RegA activation occurs through essential phospho-transfer to D212 (Thomason et al., 1999). Basal activated RegA maintains intracellular cAMP at low levels. RegA is inhibited by activated pERK2. ERK2 is auto-phosphorylated, but in a manner that is activated downstream of CAR1 (Brzostowski and Kimmel, 2006). The PPase that de-phosphorylates pERK2 is inhibited by activated CAR1. Thus, stimulation of CAR1 activates pERK2, which inhibits RegA, which allows accumulation of newly synthesized cAMP. RegA inhibition likely occurs through pERK2 phosphorylation at T676 (Adhikari et al., 2020).

#### 3.1.2 cAMP compartments

##### 3.1.2.1 Intracellular cAMP

The primary target for intracellular cAMP is the cAMP-dependent protein kinase (protein kinase A, PKA). PKA is composed of two subunits. An inhibitory regulatory subunit (PKAreg) and the kinase catalytic subunit (PKAcat). In quiescent cells, the two subunits are bound and PKAcat is inactive. Upon accumulation of intracellular cAMP, PKAreg is complexed with cAMP, leading to dissociation from and activation of PKAcat.

##### 3.1.2.2 Extracellular cAMP

cAMP-activated cells relay the signal by secreting cAMP and recruiting additional cells in tight co-ordination. The mechanism for secretion is not known, but may involve vesicle release (Kriebel et al., 2008). Spatially, cAMP is released from the cell posterior, as cells orient toward the gradient, with the anterior experiencing an initial high signal (Kriebel et al., 2008; McMains et al., 2008).

#### 3.1.3 De-activation of ACA

##### 3.1.3.1 CAR1 adaptation

Upon CAR1 binding to cAMP, G proteins dissociate, CAR1 is phosphorylated, and binding affinity to cAMP is reduced (Klein et al., 1985; Hereld et al., 1994; Janetopoulos et al., 2001; Xu et al., 2010). The reduced affinity of CAR1 for cAMP reflects reduced G protein coupling. While CAR1 is still able to engage cAMP, CAR1 is not able to transmit a signal through G proteins. Since the pathways for ACA activation require signaling *via* Gβγ, these receptors are functionally de-sensitized to cAMP activation for ACA. CAR1 adaptation leads to cessation of cAMP synthesis, termination to continued cAMP accumulation, ligand clearing (see below), and loss of CAR1 stimulation.

##### 3.1.3.2 Ligand clearing

cAMP oscillations not only require activated cAMP synthesis, but also PDE actions that return elevated cAMP levels to a basal state. PDE1 is the primary enzyme that degrades extracellular cAMP. Removal of environmental cAMP terminates all CAR1 responses, including non-adapted signaling.

##### 3.1.3.3 Transient downstream pathway feedback inhibition

Apart from receptor silencing, the ACA activation paths are subject to feedback inhibition. As stated previously, Ras proteins are activated by GEFs but de-activated by GAPs. Ras de-activation is accomplished by 2 processes. First, downstream activation of PKA feedback inhibits GEFs through the scaffold Sca1 (Charest et al., 2010). Also, while GAP activation *via* CAR1 is delayed relative to the GEFs, the parallel inactivation of GEFs and activation of GAP attenuates Ras signaling (Rappel and Firtel, 2012), including mTORC2 and PI3K. Indeed, mTORC2 activity, as measured by pPKBR1, oscillates with cAMP, pERK1, and pERK2 (see Figure 2A). PI3K also shows rapid deactivation, with PIP_3_ degraded to PIP_2_ by the PTEN phosphatase (Funamoto et al., 2002; Huang et al., 2003; Sasaki et al., 2007). PI3K oscillations are observed by the rapid membrane association/dis-association of PIP_3_-binding proteins, (*e*.*g*., CRAC), following a single cAMP stimulus (Parent et al., 1998).

Regardless of these inhibitory events, they are secondary to receptor silencing. As we have shown, cells that are adapted to cAMP response for ERK2, Ras, mTORC2, and even chemotaxis (Liao et al., 2013; Meena and Kimmel, 2017) are fully responsive to stimulation of another GPCR, Far1 (see Figure 6). Thus, the feed-back inhibitions to ACA are transient and are immediately reversable, mechanisms that are inconsistent with these as primary modes for temporally regulated oscillations.

#### 3.1.4 Intracellular cAMP degradation

##### 3.1.4.1 pERK2/pERK1/RegA circuitry

Ligand-stimulated auto-phosphorylation of pERK2 adapts similarly to that described above for ACA. However, action to the PPase is non-adaptive and is dependent on ligand clearing. Separately another path is used to re-activate the PPase.

pERK2 activates MEK1 which phosphorylates ERK1. Activated pERK1 activates de-phosphorylation of ERK2 (*e*.*g*., *via* PP2A), which de-activates ERK2. Following receptor stimulation, pERK2 is activated first, which activates pERK1, which de-activates pERK2. In the absence of active pERK2, RegA is reactivated initiating the clearance of intracellular cAMP and inactivation of PKA. There is an additional PDE for intracellular cAMP, PDE6. PDE6 is cAMP-activated and eventually degrades high level cAMP, as when RegA activity is persistently low (Bosgraaf et al., 2002).

#### 3.1.5 Target modes toward a Re-Responsive basal state

##### 3.1.5.1 PKA feedback

CAR1 activation inhibits de-phosphorylation of pERK2 leading to inhibition of RegA. With ligand clearing and activation of pERK1, the PPase is activated and pERK2 is de-phosphorylated. PPase activity must be reduced to allow re-activation of pERK2. We suggest the convergence of two actions. Clearly, pERK1 inhibition of pERK2 could feed-back inhibit MEK1/ERK1. However, we suggest that activated PKAcat is the more significant, as reduced PKAcat leads to elevated pERK1, even in the absence of pERK2. Conversely, cells with constitutively activated PKA (*i*.*e*., cells lacking PKAreg) have a diminished pERK1 response and a more rapid decline in pERK1 levels than in WT. PKA may function through MEK1, a PPase, or another mechanism.

We had also previously noted a secondary role of PKA in feedback-inhibition of Ras signaling (see 3.1.3.3 above). PKA may also play a secondary role to feedback-activate RegA (Brzostowski and Kimmel, 2006). S413 is suggested as a potential PKA site in RegA (Kuburich et al., 2019), but an S413A mutation does not phenocopy a *regA*-null mutation for developmental aggregation (Kuburich et al., 2019), whereas a D212N mutation is inactive for RegA PDE (Thomason et al., 1999).

##### 3.1.5.2 Receptor re-sensitization

Receptors are de-sensitized to a saturating or a continuous, non-varying ligand stimulus, causing both ERK2 auto-phosphorylation and ACA signaling to terminate. Following de-adaptation (see below), receptors become re-sensitized to another round of stimulation. Furthermore, the different receptors are not cross-adapted. Since effector pathways converge immediately downstream, cells adapted to one ligand (*e*.*g*., cAMP) remain sensitive to a heterologous ligand (*e*.*g*., Folate), which can re-activate ERK2, ACA, and chemotaxis. ERK2 de-phosphorylation is inhibited independently of G proteins and does not adapt to continuous stimulation. For ERK2, kinase activity will adapt in the presence of saturating ligand concentrations, but the PPase remains inactive and pERK2 is persistent. Ligand inactivation allows unbound receptors to become re-sensitized.

Re-sensitization involves de-phosphorylation and re-coupling to G proteins. This is tightly regulated temporally and is specific to each receptor. Far1 becomes re-responsive in <2 min, whereas CAR1 requires >5 min (Liao et al., 2013). This delay ensures that all intracellular pathways have become re-set to their basal state and underscores the ∼6 min oscillation rate seen for cAMP wave formation during development or in shaking culture (see Supplementary Figure S1).

### 3.2 Developmental cAMP oscillations

During *Dictyostelium* development, oscillating waves of cAMP organize cells for multi-cellular aggregation. The cross regulation of ERK2 and ERK1, integrated with input to cAMP stability, explains oscillatory cAMP wave production, cAMP signal-relay, and directed chemotactic aggregation. cAMP activates ACA and pERK2. cAMP is synthesized and stabilized through inhibition of RegA. Secreted cAMP recruits additional cells for signal-relay and aggregation, but also elicits adaptation of CAR1; ACA and ERK2 kinase are deactivated. In parallel, ERK1 is activated, which inhibits pERK2. Intracellular cAMP is cleared by the re-activated RegA and other downstream pathways return to a basal state. Extracellular cAMP is degraded by secreted PDE1 and cells become re-sensitized for another pulse of extracellular cAMP stimulation.

Thus, we have proposed an integrated model that describes how periodic cellular cycling from a basally responsive state, to activation, to de-activation, and, finally, back to a basal, re-responsive state can generate the oscillating cAMP signals that are required to direct multicellular development of *Dictyostelium*.

## 4 Materials and methods

### 4.1 Cell culture

*Dictyostelium* AX3 WT strain, *erk2*-null (Nichols et al., 2019)*, erk1*-null (Nichols et al., 2019)*, mek1*-null (Sobko et al., 2002; Mendoza et al., 2005)*, B56*-null (Lee et al., 2008)*, acaA*-null (Hirose et al., 2021), *acaA*-/*acrA*-/*acgA*-null (Hirose et al., 2021)*, erk1* ^-^/ERK1-RFP, *mek1* ^-^/GFP-MEK1, *pkaR*-null (Shaulsky et al., 1998)*, pkaC*-null (Mann et al., 1992), and *erk2* ^-^/FLAG-ERK2 cells were grown axenically in HL5 medium containing 100 μg/mL of ampicillin and 100 μg/mL streptomycin at 22°C in suspension culture, shaking at ∼180 rpm, to a density of 1–1.5 × 10^6^ cells/mL. *erk2*-*, erk1*-*, mek1*-*, B56-, acaA*-/*acrA*-/*acgA*-, and *pkaC*-null cells were grown under selection in 5 μg/mL blasticidin (InvivoGen # ant-bl). *erk1* ^-^/ERK1-RFP cells, *mek1* ^-^/GFP-MEK1 cells, and *erk2* ^-^/FLAG-ERK2 cells were grown under selection in 10 μg G418 (Gold Biotechnology # G-418-5). Cell lines are available at dictyBase (Basu et al., 2013; Fey et al., 2013).

### 4.2 cAMP pulsing

Cells were grown to a density of 1–1.5 × 10^6^ cells/mL. For development, cells were washed twice with developmental buffer [DB (Meena and Kimmel, 2016)] at 22°C and resuspended in DB to a density of 2 × 10^7^ cells/mL. The suspension was gently shaken at 30–40 rpm at 22°C. A time-controlled peristaltic pump was used to rapidly (<20 s) add cAMP to 75 nM at 6 min intervals for 5 h. Pulsing was stopped and cells were shaken for an additional 12 min. To deactivate ACA, cells were washed and resuspended in DB containing 3 mM caffeine (freshly prepared). After 20 min, cells were washed and resuspended in DB (Meena and Kimmel, 2016). *acaA*-null and *acaA-*/*acrA*-/*acgA*-null cells were not treated with caffeine.

### 4.3 cAMP stimulation

cAMP developed cells at 2 × 10^7^ cells/mL were shaken for 30 s, and then stimulated with a bolus of cAMP (Meena and Kimmel, 2016). To monitor the kinetics for immunoblotting (see below), a 75 μL of sample was added to 25 μL (4x) SDS lysis buffer. For Jess Protein Analysis (see below), 20 μL of the sample was mixed with 80 μL of RIPA buffer supplemented with PhosStop and Protease inhibitors.

### 4.4 Folate stimulation

Cells grown at a density of 1–1.5 × 10^6^ cells/mL were washed twice and resuspended in DB and shaken for 30–90 min at 22°C. Cells were then stimulated with a bolus of folate. Samples for immunoblotting were collected as above, for cAMP.

### 4.5 Immunoblotting

pERK1, pERK2, pPKBR1, FLAG-ERK2, GFP-MEK1, and actin were monitored in whole cell lysates by immunoblotting following gel electrophoresis (Bio-Rad, 4%–15% tris glycine gels), with antibodies against human pERK^T202/Y204^ [1:1000, Cell Signalling # 9101 for pERK1 and pERK2], human p70S6K^T389^ [1:1000; Cell signaling #9205 for pT470-PKBR1], RFP [1:1000; chromotek # 6G6], GFP [1:1000; Chromotek # pabg1], FLAG [1:1000; Sigma-Aldrich #F3165], and actin [Santa Cruz Biotechnology # SC-1616] (Meena and Kimmel, 2016; Jaiswal and Kimmel, 2019). We validated pERK1, pERK2, and actin immunoblots using Jess Protein Simple (Bio-Techne). A 1:50 dilution of pERKT202/Y204 and a 1:100 dilution of actin-HRP were prepared. For the experiment, 2 μg of protein was loaded into a well in the cartridge, and the remaining steps, including loading other reagents, analysis, and image processing, were carried out according to the manufacturer’s instructions. All immunoblot experiments were replicated at least 3 times, with data shown for both cAMP and folate, unless otherwise described.

## Data Availability

The original contributions presented in the study are included in the article/Supplementary Material, further inquiries can be directed to the corresponding author.
