# Supplementary figures and images for: An integrated, cross-regulation pathway model involving activating/adaptive and feed-forward/feed-back loops for directed oscillatory cAMP signal-relay/response during the development of Dictyostelium
Source: Front Cell Dev Biol. 2024 Jan 31;11:1263316. doi: 10.3389/fcell.2023.1263316 (PMC10865387; doi:10.3389/fcell.2023.1263316)

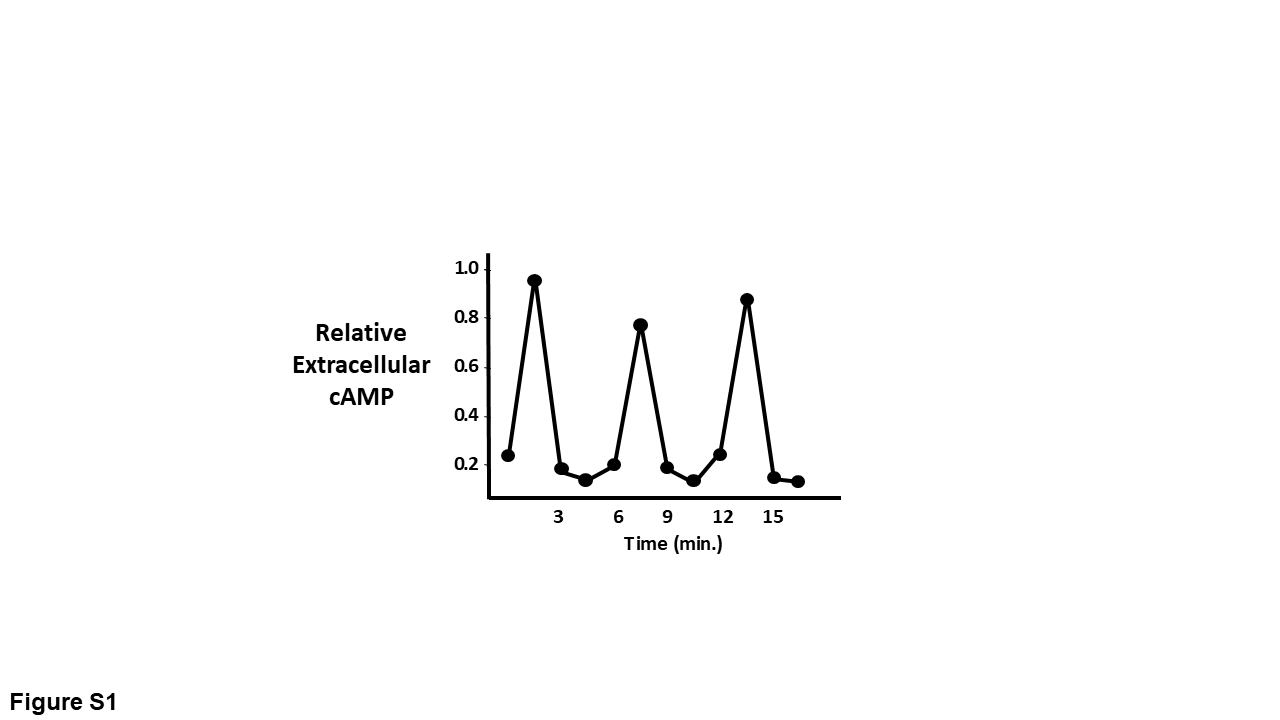

Supplement: Supplementary file 1 [file Presentation1.zip › Suppl. Figure S1.TIF]

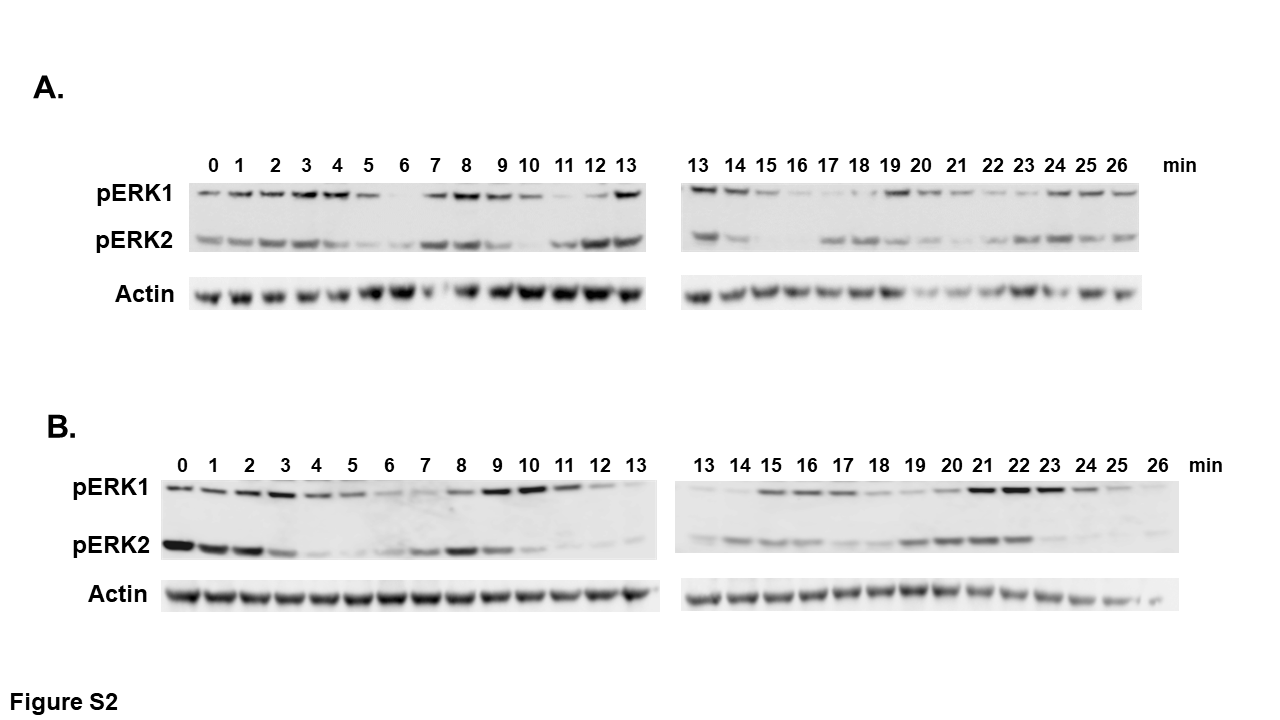

Supplement: Supplementary file 1 [file Presentation1.zip › Suppl. Figure S2 A, B.TIF]

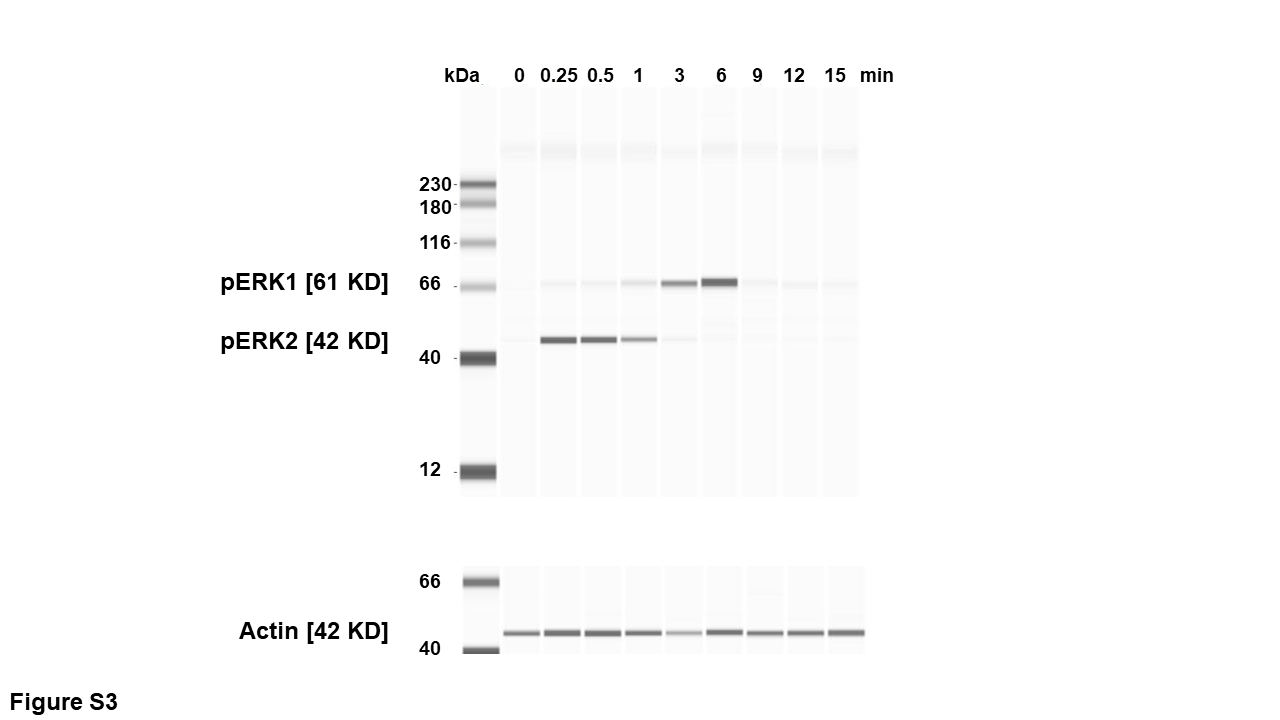

Supplement: Supplementary file 1 [file Presentation1.zip › Suppl. Figure S3.TIF]

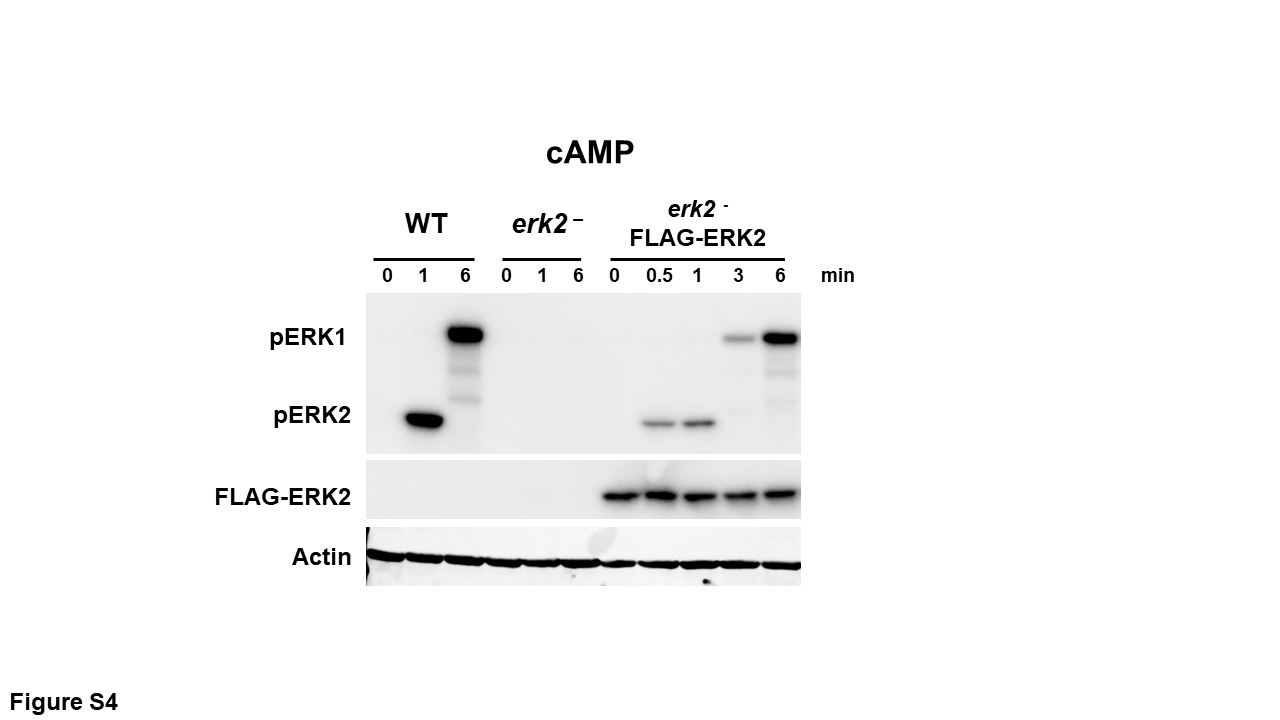

Supplement: Supplementary file 1 [file Presentation1.zip › Suppl. Figure S4.TIF]

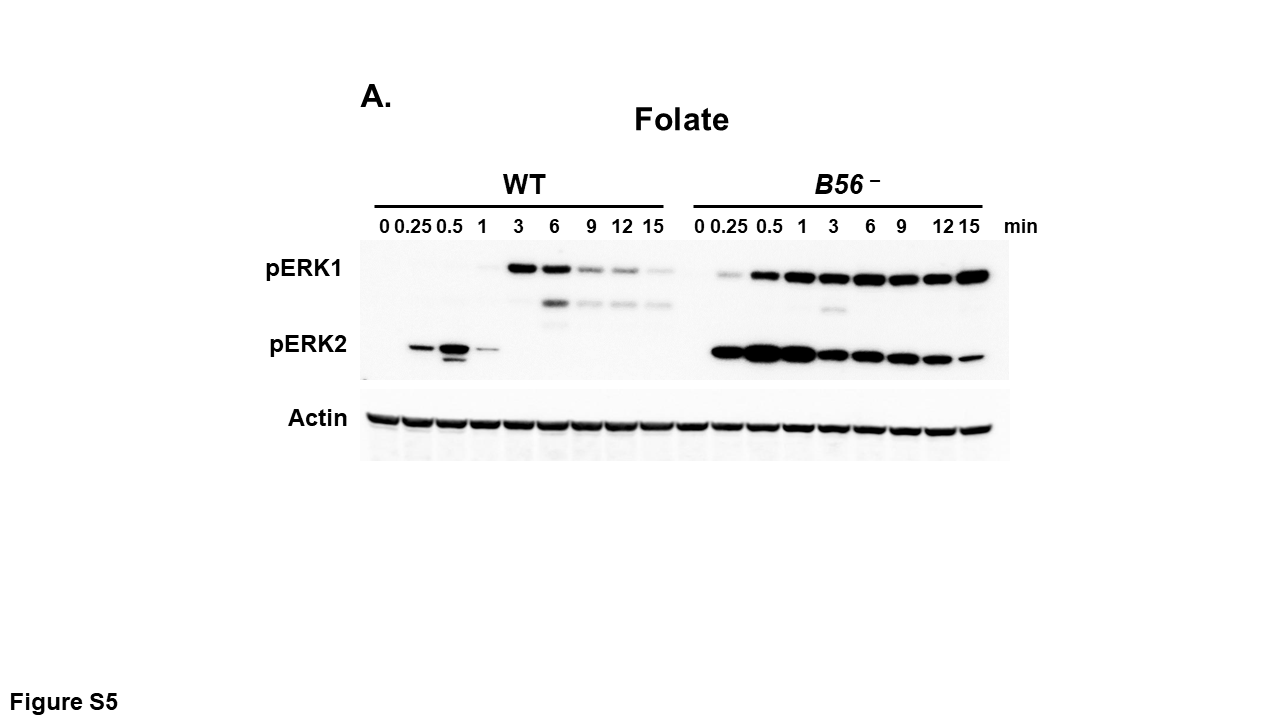

Supplement: Supplementary file 1 [file Presentation1.zip › Suppl. Figure S5A.TIF]

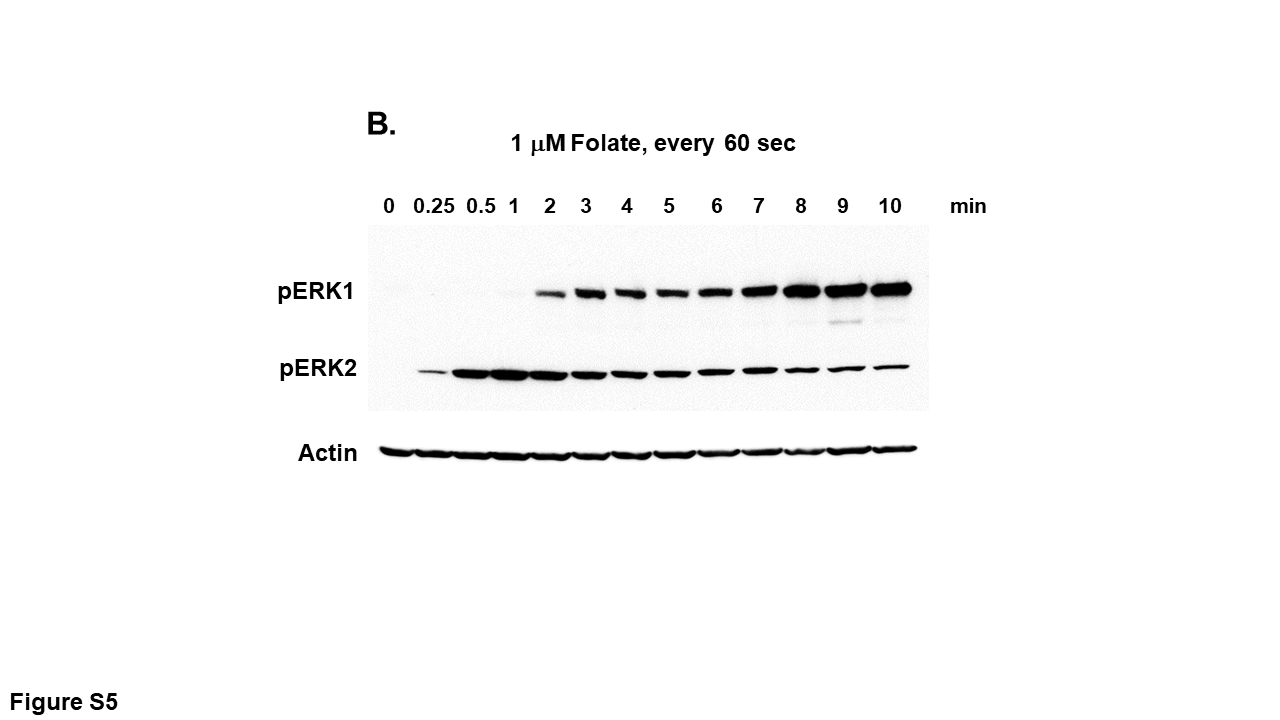

Supplement: Supplementary file 1 [file Presentation1.zip › Suppl. Figure S5B.TIF]

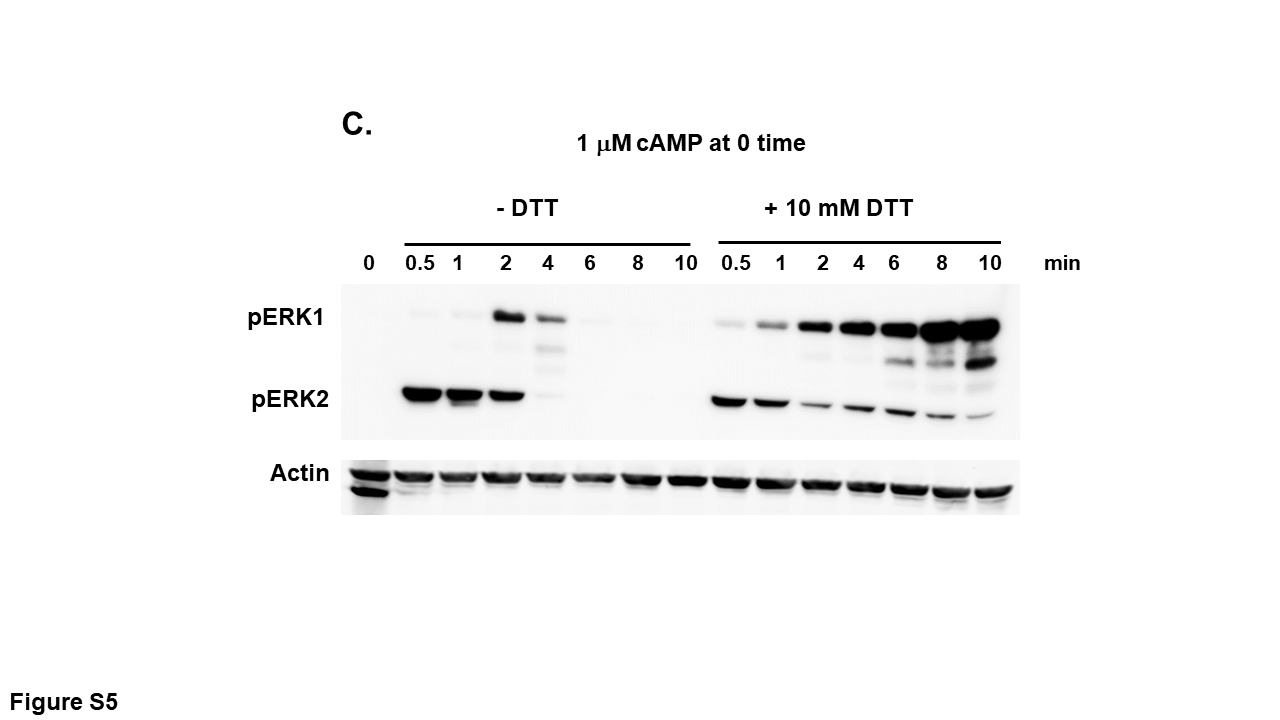

Supplement: Supplementary file 1 [file Presentation1.zip › Suppl. Figure S5C.TIF]

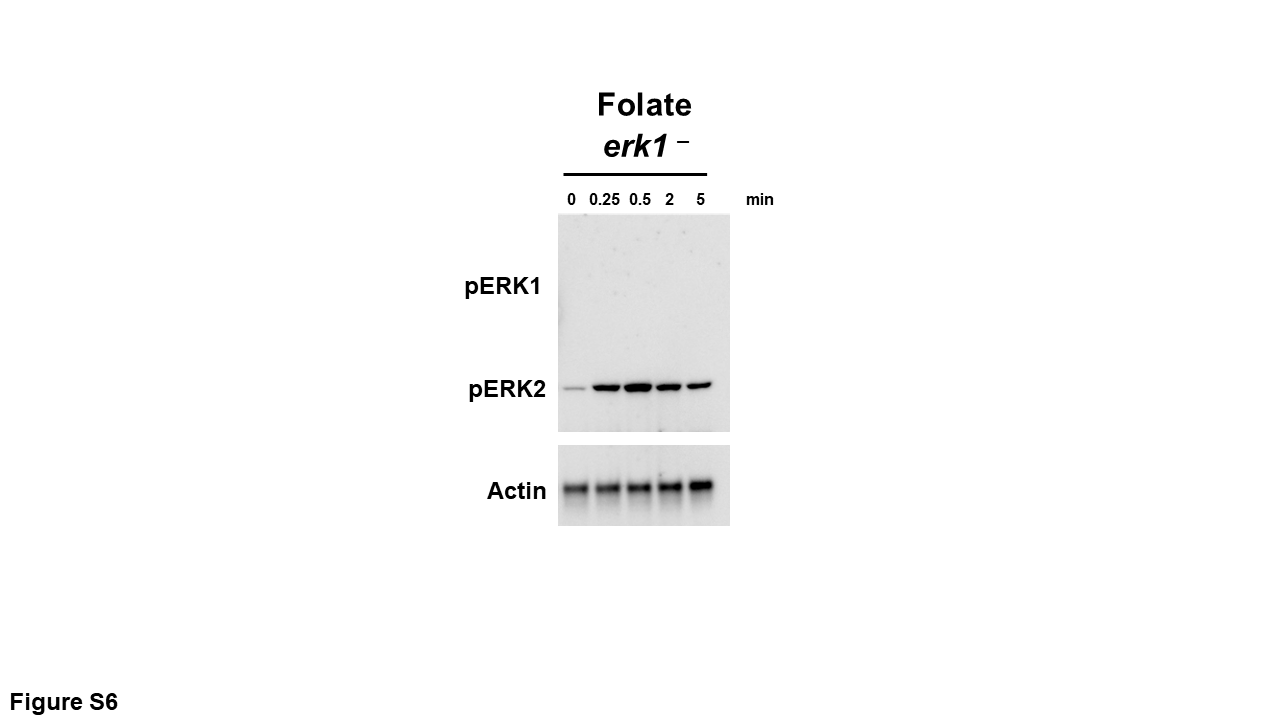

Supplement: Supplementary file 1 [file Presentation1.zip › Suppl. Figure S6.TIF]

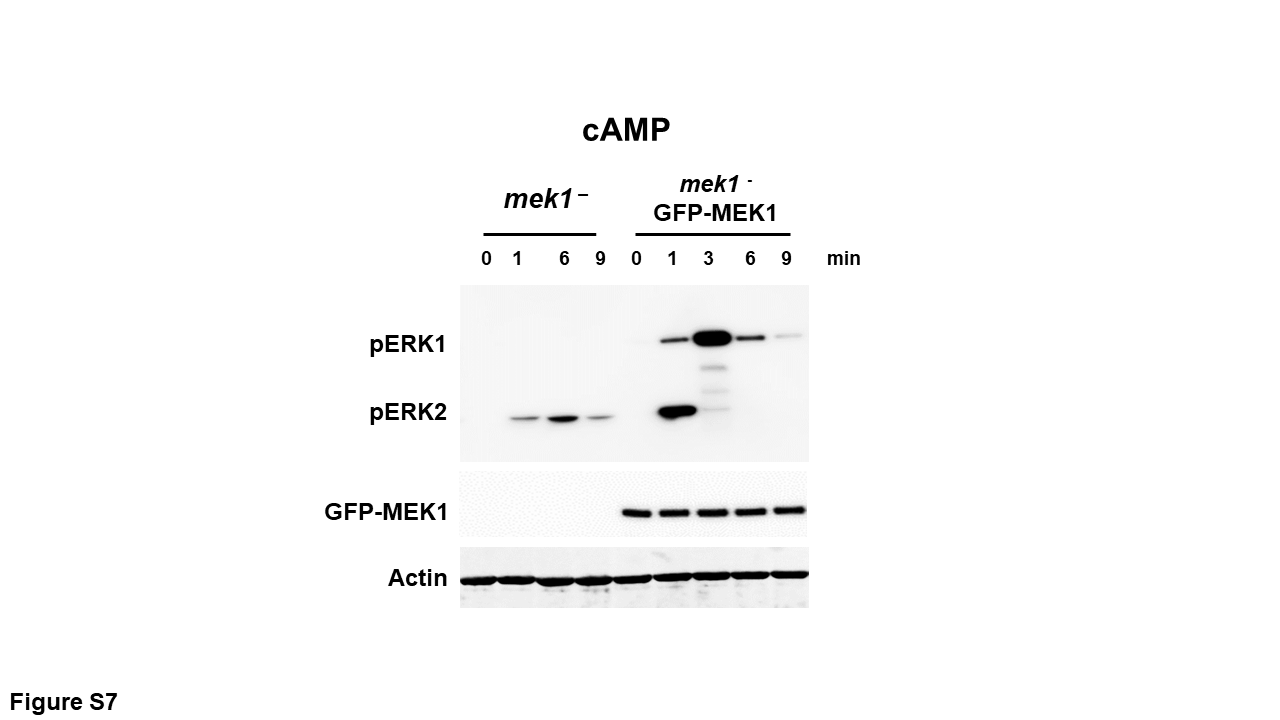

Supplement: Supplementary file 1 [file Presentation1.zip › Suppl. Figure S7.TIF]

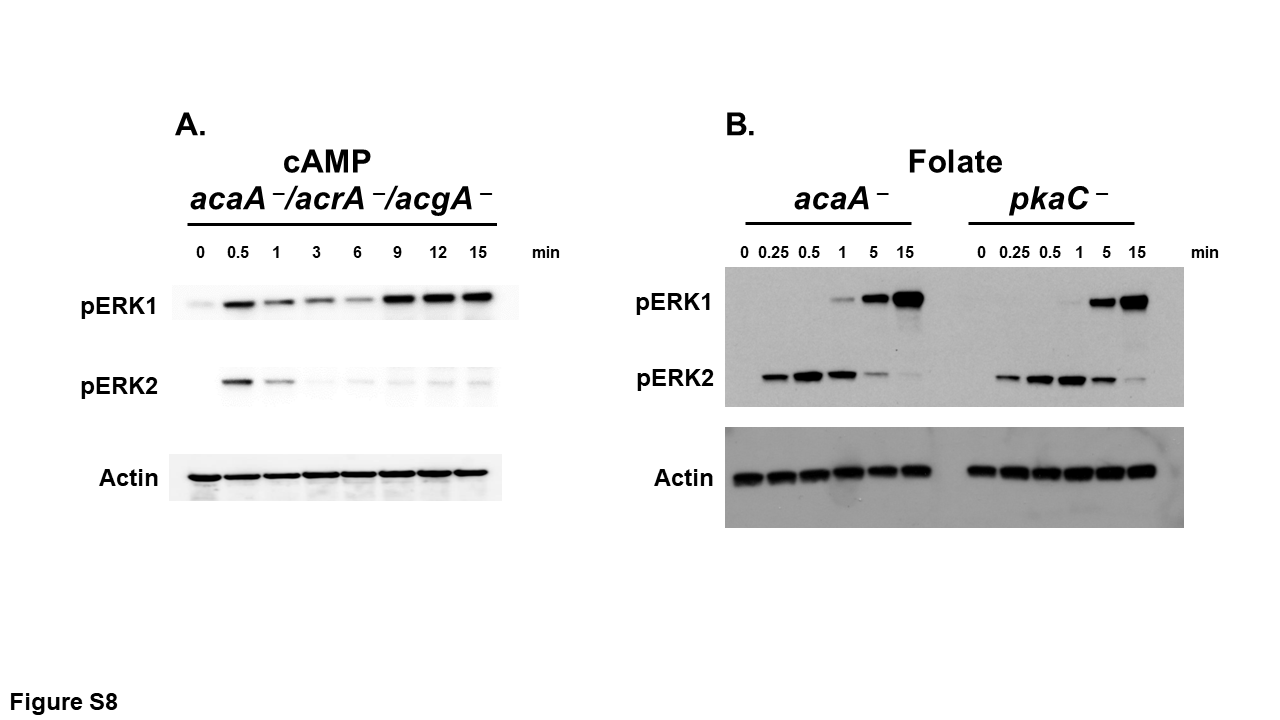

Supplement: Supplementary file 1 [file Presentation1.zip › Suppl. Figure S8 A, B.TIF]
