# Supplementary material for: An integrated, cross-regulation pathway model involving activating/adaptive and feed-forward/feed-back loops for directed oscillatory cAMP signal-relay/response during the development of Dictyostelium
Source: Front Cell Dev Biol. 2024 Jan 31;11:1263316. doi: 10.3389/fcell.2023.1263316 (PMC10865387; doi:10.3389/fcell.2023.1263316)
Supplement: Supplementary file 2 [file Image1.pdf]

SUPPLEMENTAL FIGURE LEGENDS

Supplemental Figure S1. Spontaneous oscillations of cAMP.

Data show relative levels of extracellular cAMP in shaking culture, indicating spontaneous cAMP oscillations [as adapted from (Kimmel, 1987;McMains et al., 2008)].

Supplemental Figure S2. Spontaneous oscillations of ERK1 and ERK2 phosphorylation.

Cells were pulsed to a final concentration of 75 nM cAMP every 6 min for 5 hr. Cells were washed, resuspended in fresh buffer, and incubated for 30 min without exogenous cAMP to allow spontaneous cAMP oscillations. Aliquots were collected at 1-min intervals and ERK1 and ERK2 phosphorylations assayed by immunoblot. **A. and B.** represent two different data sets. Figure S2B data are quantified for Figure 2A.

Supplemental Figure S3. Time course for cAMP-stimulated phosphorylation of ERK1 and ERK2.

Cells were pulsed to a final concentration of 75 nM cAMP every 6 min for 5 hr, and then stimulated with 10 μM cAMP at time 0. Aliquots were collected at indicated times and ERK1 and ERK2 phosphorylations and actin levels assayed by immunoblot. Please note that these data were assayed using the JESS instrument and so the output has a different appearance to other immunoblots (*e.g*. Figure 2B).

Supplemental Figure S4. FLAG-ERK2 rescues phosphorylation of ERK2 and ERK1 in *erk2*-null cells.

A FLAG-ERK2 fusion was expressed in *erk2*-null cells. WT, *erk2*-null cells and *erk2*-null cells expressing FLAG-ERK2 were pulsed to a final concentration of 75 nM cAMP every 6 min for 5 hr, and then stimulated with 10 μM cAMP at time 0. Aliquots were collected at indicated times and ERK1 and ERK2 phosphorylations, FLAG-ERK2, and actin levels assayed by immunoblot.

Supplemental Figure S5.

A. pERK1 is extended in folate-stimulated cells lacking the PPase for pERK2.

WT and *B56*-null cells were shaken in DB culture for 90 min and stimulated with 1 μM folate. Aliquots were collected at indicated times and ERK1 and ERK2 phosphorylations and actin levels assayed by immunoblot.

B. Inhibition of pERK2 de-phosphorylation extends pERK1 in folate-stimulated WT cells.

WT cells were shaken in DB culture for 90 min and stimulated with 1 μM folate and then with another 1 μM folate every min thereafter. Aliquots were collected at indicated times and ERK1 and ERK2 phosphorylations and actin levels assayed by immunoblot.

C. Inhibition of pERK2 de-phosphorylation by persistent CAR1 stimulation extends pERK1 in WT cells.

WT cells were pulsed to a final concentration of 75 nM cAMP every 6 min for 5 hr, and then stimulated with 10 μM cAMP at time 0. One set of cells received no further

treatment; one set of cells included 10 mM DTT. Aliquots were collected at indicated times and ERK1 and ERK2 phosphorylations and actin levels assayed by immunoblot.

Supplemental Figure S6. ERK1 is required for pERK2 de-phosphorylation in folate-stimulated cells.

erk1-null cells were shaken in DB culture for 90 min and stimulated with 1 μM folate at time 0. Aliquots were collected at indicated times and ERK1 and ERK2 phosphorylations and actin levels assayed by immunoblot.

Supplemental Figure S7. GFP-MEK1 expression rescues phosphorylation kinetics of ERK2 and ERK1 in *mek1*-null cells.

A GFP-MEK1 fusion was expressed in *mek1*-null cells. *mek1*-null cells and *mek1*-null cells expressing GFP-MEK1 (*mek1*$^{-}$/GFP-MEK1) were pulsed to a final concentration of 75 nM cAMP every 6 min for 5 hr, and then stimulated with 10 μM cAMP at time 0. Aliquots were collected at indicated times and ERK1 and ERK2 phosphorylations, GFP-MEK1, and actin levels assayed by immunoblot.

Supplemental Figure S8. Loss of cAMP/PKA signaling inhibits de-phosphorylation of pERK1.

A. Absence of intracellular cAMP inhibits de-phosphorylation of pERK1.

WT and *acaA-/acgA-/acrA*-null cells were pulsed to a final concentration of 75 nM cAMP every 6 min for 5 hr, and then stimulated with 10 μM cAMP at time 0. Aliquots were

collected at indicated times and ERK1 and ERK2 phosphorylations and actin levels assayed by immunoblot.

B. Diminished intracellular cAMP/PKA signaling inhibits de-phosphorylation of pERK1 in folate-stimulated cells.

acaA-null and *pkaC*-null cells were shaken in DB culture for 90 min and stimulated with 1 μM folate at time 0. Aliquots were collected at indicated times and ERK1 and ERK2 phosphorylations and actin levels assayed by immunoblot.

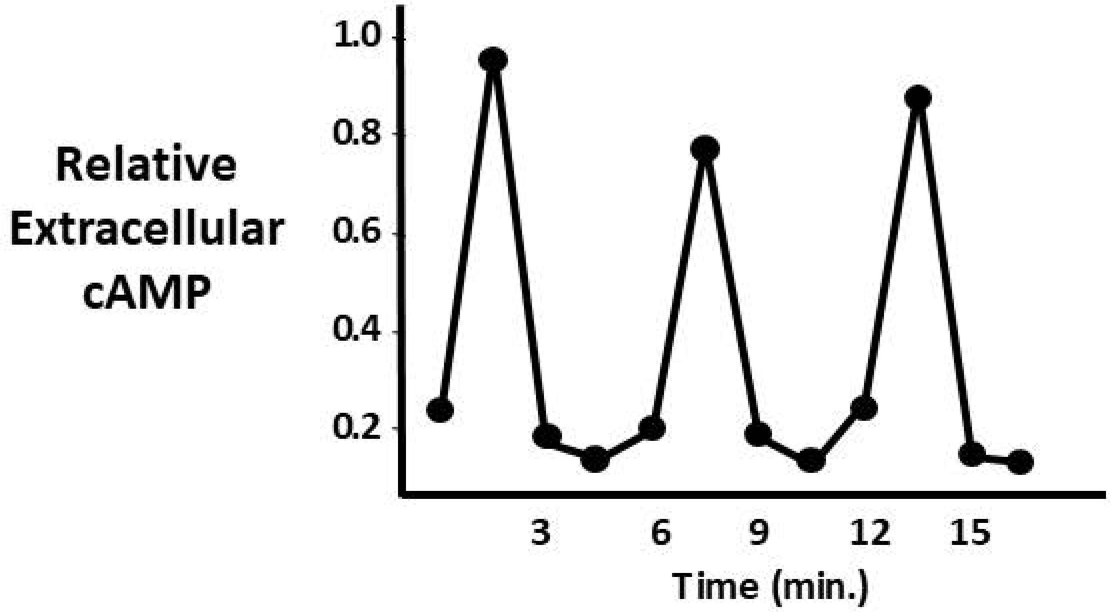

Figure S1

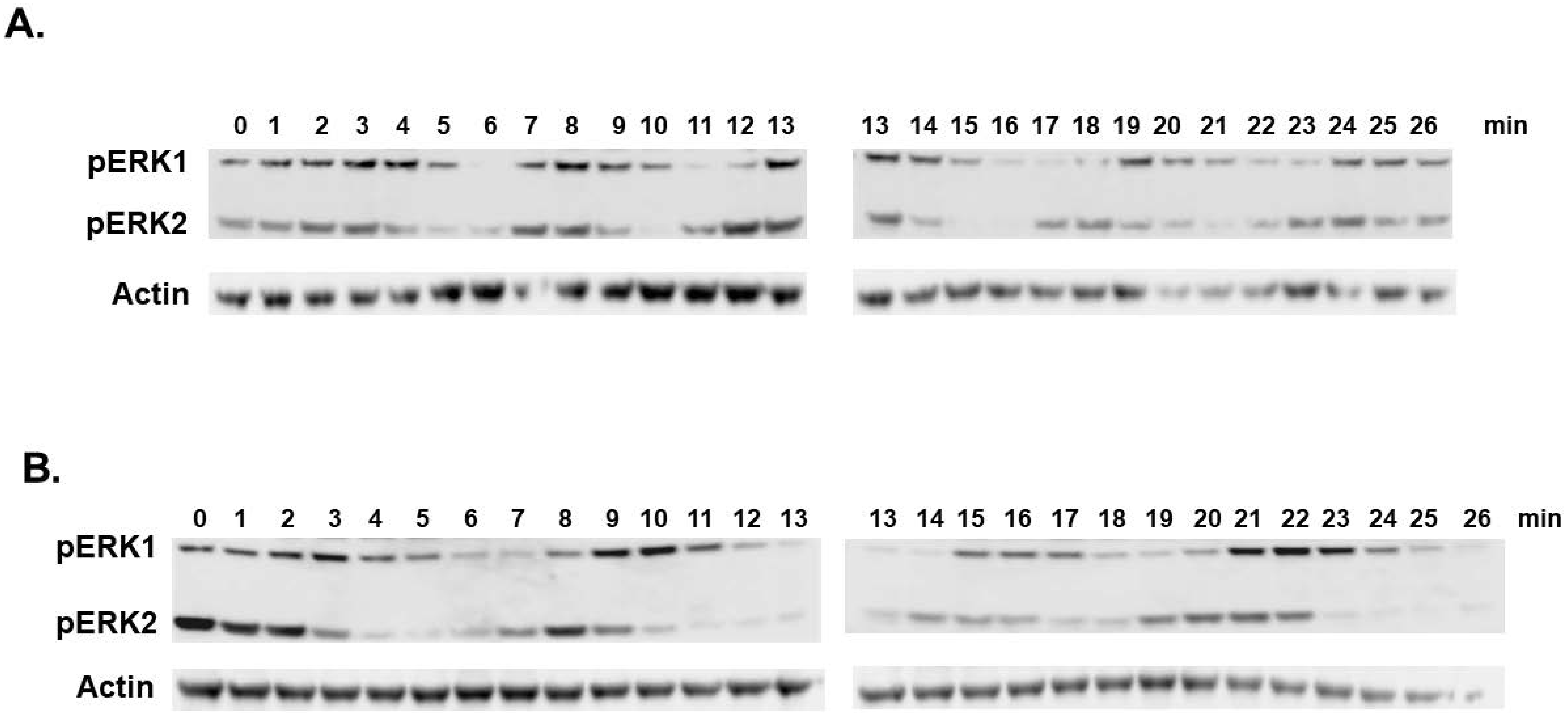
A.
0 1 2 3 4 5 6 7 8 9 10 11 12 13
13 14 15 16 17 18 19 20 21 22 23 24 25 26 min
pERK1
pERK2
Actin
B.
0 1 2 3 4 5 6 7 8 9 10 11 12 13
13 14 15 16 17 18 19 20 21 22 23 24 25 26 min
pERK1
pERK2
Actin

Figure S2

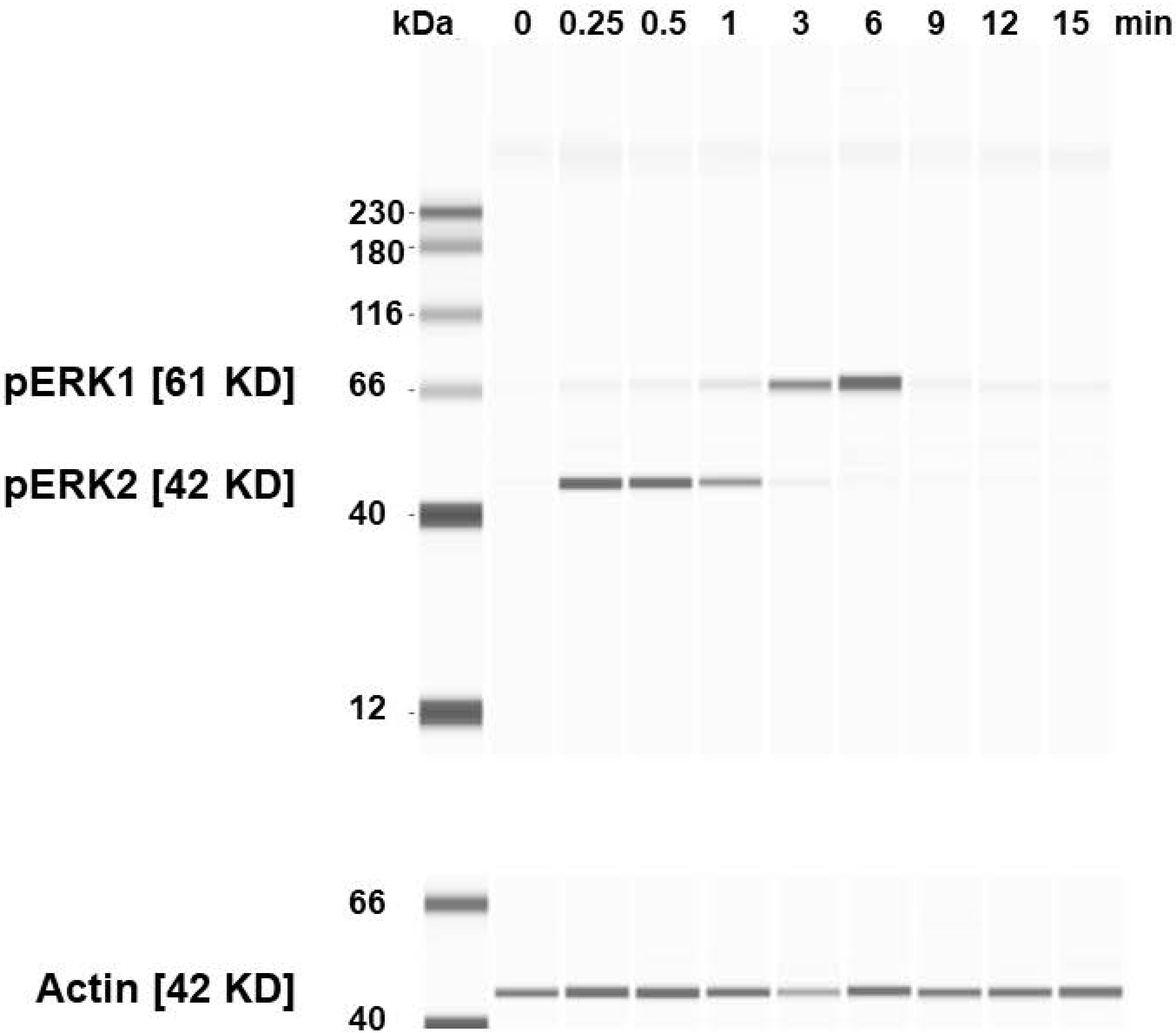
kDa
0
0.25
0.5
1
3
6
9
12
15
min
230
180
116
66
40
12
pERK1 [61 KD]
pERK2 [42 KD]
66
40
Actin [42 KD]

Figure S3

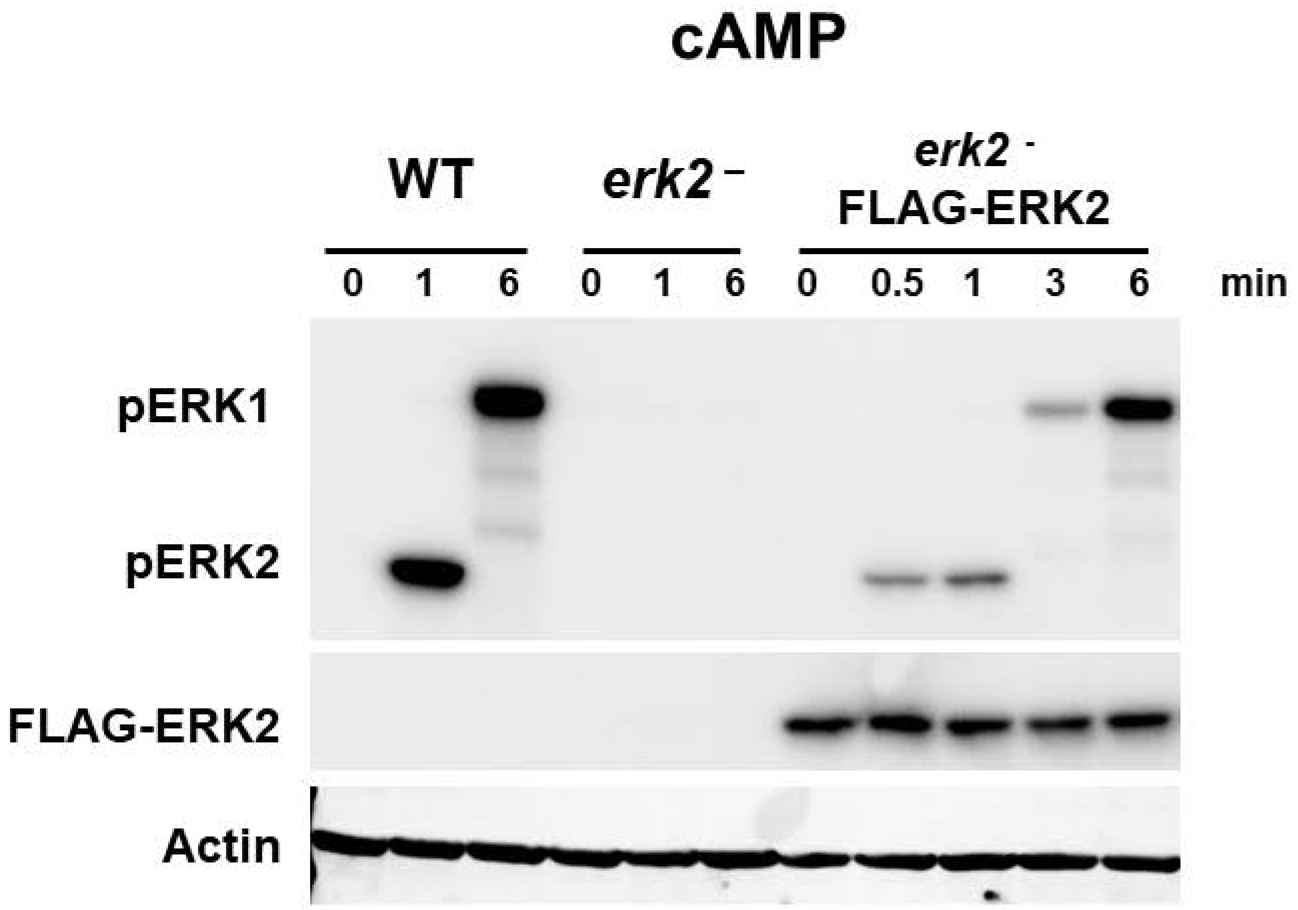
cAMP
WT
erk2⁻
erk2⁻ FLAG-ERK2
0 1 6
0 1 6
0 0.5 1 3 6
min
pERK1
pERK2
FLAG-ERK2
Actin

Figure S4

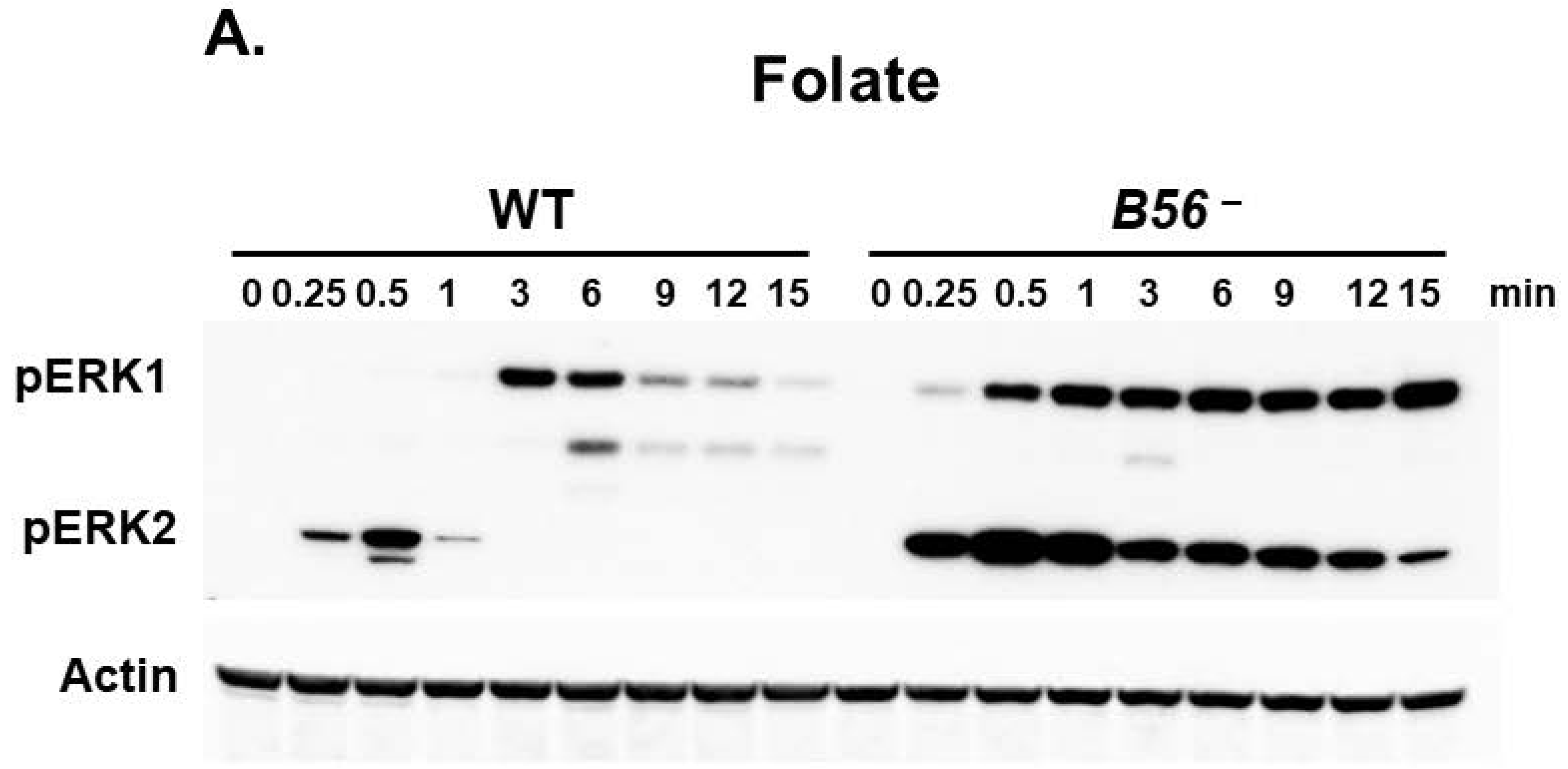
A.
Folate
WT
B56 –
0 0.25 0.5 1 3 6 9 12 15
0 0.25 0.5 1 3 6 9 12 15
min
pERK1
pERK2
Actin

Figure S5

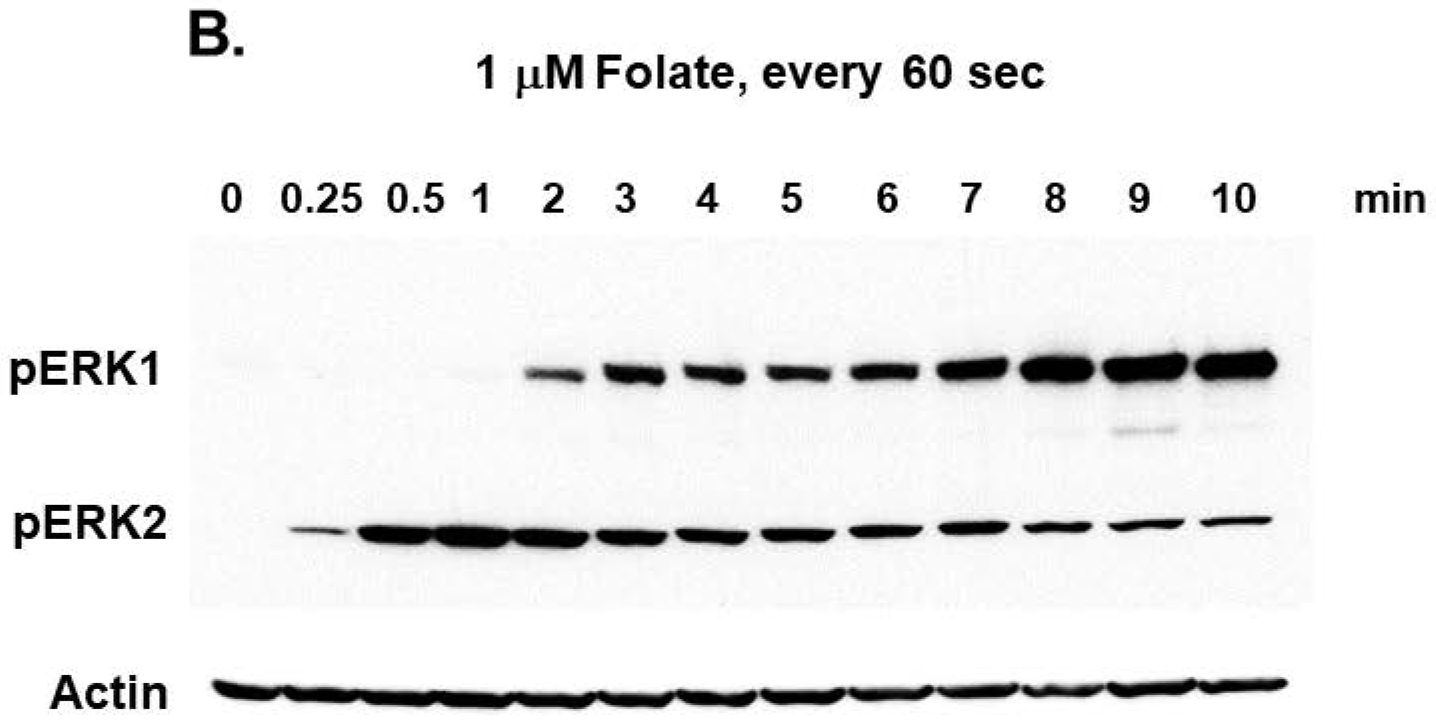
B.
1 μM Folate, every 60 sec
0 0.25 0.5 1 2 3 4 5 6 7 8 9 10 min
pERK1
pERK2
Actin

Figure S5

C.

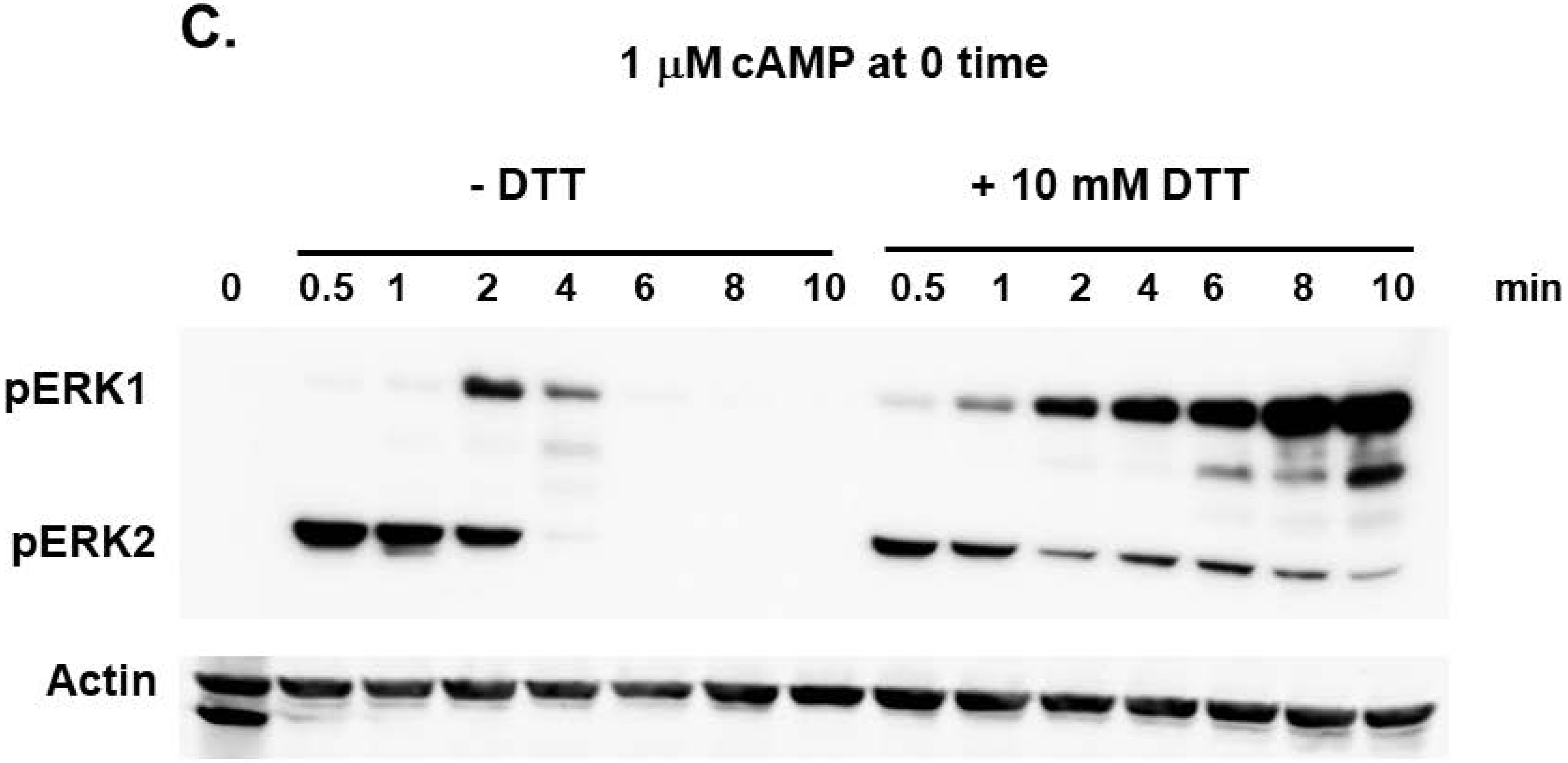

1 μM cAMP at 0 time
- DTT
+ 10 mM DTT
0
0.5
1
2
4
6
8
10
0.5
1
2
4
6
8
10
min
pERK1
pERK2
Actin

Figure S5

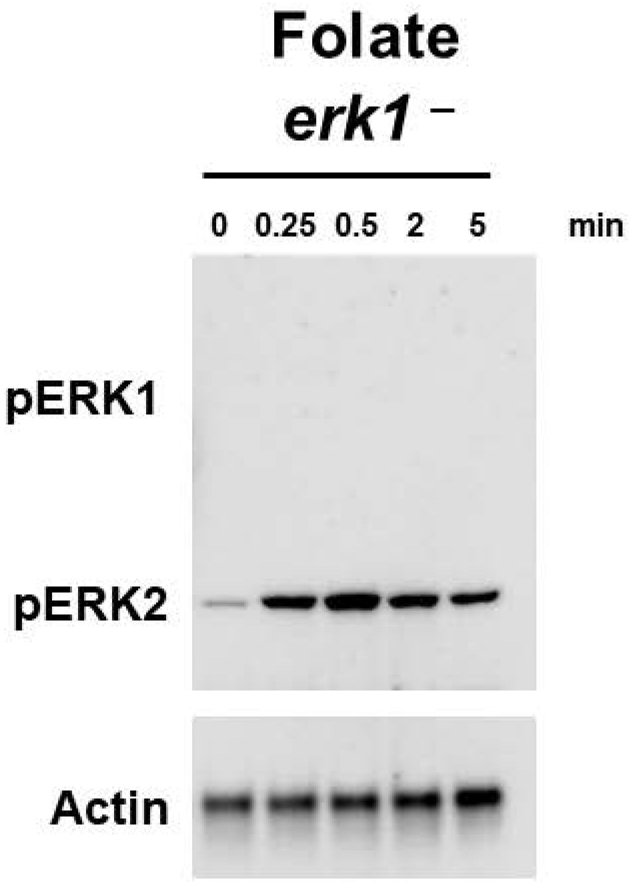

Figure S6

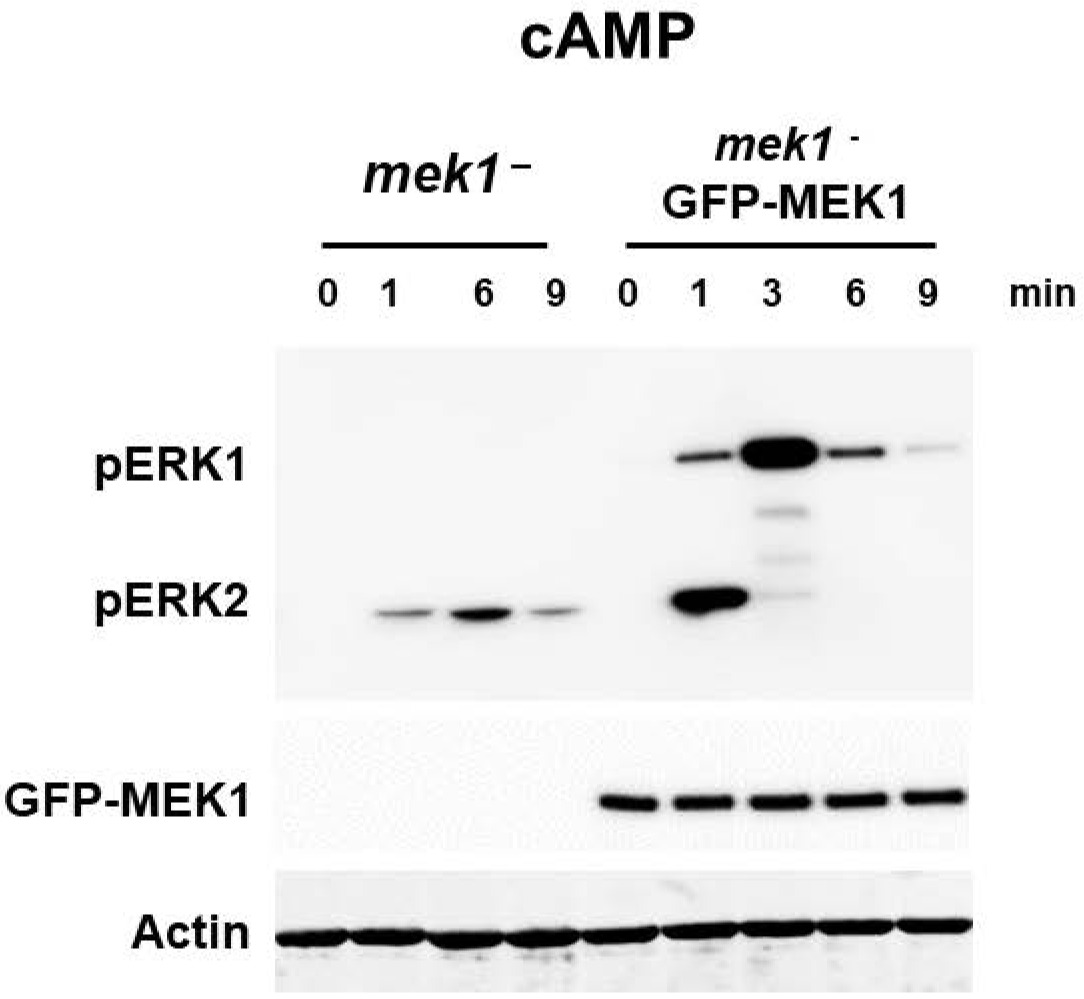
cAMP
mek1⁻
mek1⁻
GFP-MEK1
0 1 6 9 0 1 3 6 9 min
pERK1
pERK2
GFP-MEK1
Actin

Figure S7

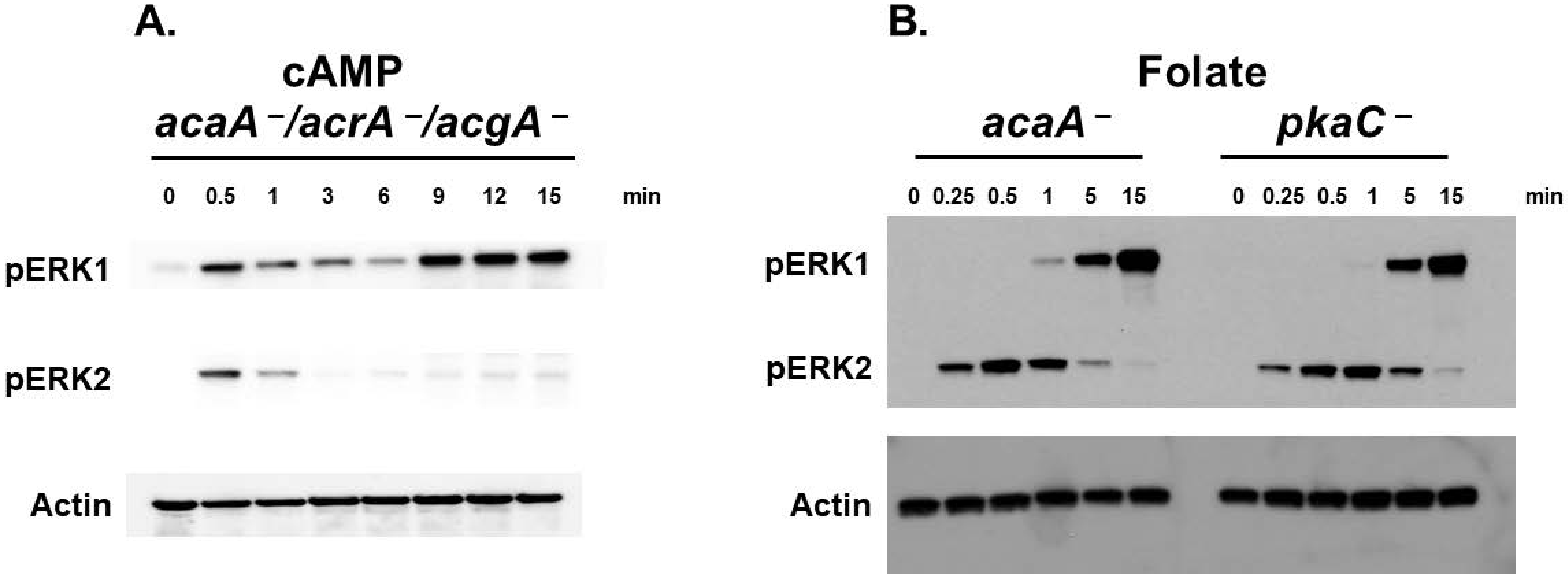
A.
cAMP
acaA⁻/acrA⁻/acgA⁻
0
0.5
1
3
6
9
12
15
min
pERK1
pERK2
Actin
B.
Folate
acaA⁻
pkaC⁻
0
0.25
0.5
1
5
15
0
0.25
0.5
1
5
15
min
pERK1
pERK2
Actin

Figure S8
